# Stranger to my face: Top-down and bottom-up effects underlying prioritization of images of one’s face

**DOI:** 10.1371/journal.pone.0235627

**Published:** 2020-07-09

**Authors:** Mateusz Woźniak, Jakob Hohwy

**Affiliations:** 1 Cognition and Philosophy Lab, Department of Philosophy, Monash University, Melbourne, Australia; 2 Social Mind Center, Department of Cognitive Science, Central European University, Budapest, Hungary; University of Amsterdam, NETHERLANDS

## Abstract

Recent studies suggest that we rapidly and effortlessly associate neutral information with the self, leading to subsequent prioritization of this information in perception. However, the exact underlying processes behind these effects are not fully known. Here, we focus specifically on top-down and bottom-up processes involved in self-prioritization, and report results from three experiments involving face detection, using a sequential match-non-match task. Across the three experiments we asked participants to associate an unfamiliar face with the self (Experiment 1), to associate one’s face with a stranger’s name (Experiment 2), and to establish both associations simultaneously (Experiment 3). We found that while participants showed evidence of bottom-up prioritization of their real faces, they did not show such an effect for self-associated strangers’ faces. However, the participants showed a robust self-related top-down effect; when presented with a self-related cue, they were later faster at classifying both subsequent correct and incorrect targets. Together, our results suggest that self-prioritization is underpinned by distinct top-down and bottom-up processes. We discuss our findings in the context of the proposal that the self acts as an “integrative glue”, and suggest an interpretation of our results within the framework of predictive coding.

## Introduction

Self-related information is preferentially processed compared to other kinds of information. This includes faster and more accurate perception, as illustrated by tasks involving perception of one’s face [1–7] or name [1, 3, 8, 9]. Self-related information benefits also in memory-related processing [10–18]. Recent studies have provided evidence that self-related effects can be observed not only for information conventionally associated with the self, such as picture of one’s face, but also for neutral stimuli which have been arbitrarily associated with the self. The first direct evidence for this effect was observed in a study by Sui, He & Humphreys [19] (but see also: [20, 21]), where participants were told to associate geometrical shapes (triangle, square, and circle) with three identities: self, friend, and stranger, represented by relevant labels. In a subsequent match-non-match task, participants had to judge whether a pairing of a shape and a label was matching or mismatching. The results showed that if the pairings were matching, then participants were faster to judge self-associated than friend- or stranger-associated stimuli, suggesting that participants demonstrated a “self-prioritization effect” for arbitrary rapidly self-associated geometrical shapes. This effect was later replicated with geometrical shapes [22–34], other kinds of visual stimuli, such as Gabor patches [35], tilted lines [36], images of food [37], avatars [38], and faces [20, 39], as well as with stimuli in other sensory modalities [40–42].

These results may suggest that there is no difference between self-related prioritization of familiar self-associated stimuli (such as one’s face or one’s name) and arbitrary self-associated ones (such as a geometrical shape or an avatar face), and that the same neurocognitive mechanism underlies both of them. However, a recent study by Woźniak, Kourtis and Knoblich [43] suggests that the picture is more complex (see also: [20]). They used a sequential version of the match-non-match task (cf. [32]) in which participants had to judge whether a face and a label are matching or not, but the stimuli were presented sequentially (1.5 second apart) and not simultaneously: first a face and then a label in Experiment 1, and first label and then a face in Experiment 2. They found that arbitrary self-association of neutral faces led to faster reaction times, if a face was presented 1.5 seconds before a label. This finding seems to be in agreement with previous results showing that neutral stimuli which are rapidly associated with the self can lead to facilitation of cognitive processing. However, they did not find that self-associated faces lead to improved cognitive processing when such faces are presented as a second stimulus, 1.5 second after a label, suggesting that they may not be prioritized. A similar pattern of results was present for the labels. It is not clear how to reconcile these apparently contradictory findings.

These findings may suggest that there is more than one cognitive process underlying the effects caused by self-association. We propose that the results from the sequential match-non-match task may be interpreted as reflecting a functional distinction between top-down and bottom-up processing. Specifically, the first stimulus in a sequence can be read as a cue telling participants which target (second stimulus) they should detect, and leading to a cascade of preparatory, and therefore top-down, processes for target detection. Because the cue was presented for only 200ms, 1.5s before the target, any influence that it had on reaction times in response to the target must have been through top-down rather than bottom-up processes, because the latter are present only during early stages of perceptual processing–typically within the first 300ms (for letter perception: [44]; for a discussion of time course of visual perception see e.g.: [45]). Indeed, it has been argued elsewhere that such a cueing task can be used to distinguish between distinct top-down and bottom-up effects, with properties of the cue influencing top-down processes, and properties of the target influencing the bottom-up processes [46]. If a reaction (accuracy, reaction time) is modulated by the identity of the cue displayed long enough before the target, irrespective of the identity of the target, then the only way in which it can have such an effect is through top-down processes, because at that time any bottom-up effects should already be extinguished. At the same time, an immediate response to the target can be driven by a combination of top-down and bottom-up effects, with properties of the target reflecting the bottom-up influence, through either stronger stimulus strength (e.g. louder, brighter stimulus) or stronger internal representation of the target (e.g. when it is easier to rapidly detect a symbol from known than unknown alphabet).

Understood this way, the Woźniak et al. [43] study suggests that the effect of self-association on reaction times may be driven exclusively by top-down mechanisms, as faster reaction times were observed only for self-related cues and not for self-related targets. However, this conclusion stands in stark contrast with classical findings showing that self-related stimuli, such as one’s own face and name, are automatically attracting attention and are processed preferentially in a bottom-up manner [8, 47–49]. Here, we describe three experiments with a sequential matching task, in which we investigated top-down and bottom-up contributions to self-prioritization by independently manipulating properties of cues and targets. We investigated faces because they allowed us to compare bottom-up processing of arbitrary self-associated stimuli (self-associated stranger’s face) with bottom-up processing of established self-related stimuli (participant’s real face).

Across the three experiments, we asked participants to associate a stranger’s face with the self (Experiment 1), one’s real face with a stranger’s name (Experiment 2), and to do both simultaneously (Experiment 3). By doing this, we intended to characterize top-down and bottom-up mechanisms responsible for self-prioritization effect in the match-non-match task, and reconcile results from recent studies investigating this effect with classical findings about perception of one’s own face.

## Experiment 1

The first experiment had two goals. First, to corroborate the pattern of results observed by Woźniak et al. [43]; second, to test whether the top-down effect in [43] can be observed in a task in which knowledge about the identity of a cue cannot induce specific perceptual expectations in participants.

For the first goal, the experiment tested whether presentation of a self-associated unfamiliar face as a target leads to faster reaction times regardless of the self- or stranger-association of a cue; such a finding would display a processing advantage similar to perception of one’s real face as reported in the literature (e.g. [3, 5, 6, 50]). This would provide evidence of self-related facilitation of bottom-up processing, independently of any top-down cue-induced mechanisms. Alternatively, no effect in reaction times would corroborate the findings from [43] where this effect was not observed.

Our second goal was to determine the influence of perceptual expectations on the previously reported top-down effect. The effect of perceptual expectations can be observed when a cue conveys (statistical) information about the identity of the target. It has been experimentally shown in studies on statistical learning that humans are sensitive to this kind of statistical structure (transitional probabilities) in sequences of stimuli [51–53]. In the study by Woźniak et al. participants associated three identities (self, friend, stranger) with three faces. However, in the matching task half the trials were matching and half were mismatching. This means that if the cue was associated with the self, then there was 50% probability that the target was associated with the self, and only 25% percent that it was associated with each one of the other two identities, i.e. friend or stranger. Thus, it is possible that their top-down effect was driven not by self-association of the cue *per se*, but by the fact that the self-associated cue informed participants that there was increased probability that the target would be a self-associated stimulus, and this perceptual expectation was driving the subsequent facilitation of processing. It is important to differentiate perceptual expectations from a response bias: while perceptual expectations reflect information about the identity of a next stimulus in a sequence, a response bias reflects expectations about which response will be correct after a target is presented. Therefore, Experiment 1 sought to determine whether the top-down self-prioritization effect will persist in a matching task in which identity of the cue does not cause unbalanced perceptual expectations about the identity of the target. In order to do this we modified the match-non-match task from Experiment 2 from [43]. In the present task, participants were first presented with a label representing one of three identities, and then, after a short delay, with one of three faces associated with these identities. Crucially, in our task there was a 1/3 probability of seeing each face after presentation of each label. We hypothesized that, if the self-prioritization effect from [43] was caused only by the fact that the self-related cue increased perceptual expectations for a self-associated target, then the self-prioritization effect should disappear. Conversely, if the effect is present in our version of the task then it cannot be attributed to perceptual expectations and it has to reflect the effect of presentation of a self-associated cue and subsequent preparatory activity to detect a self-associated target.

The fact that we used the design in which each cue was followed by each target 33% of the time had important additional consequences. Most importantly, it allowed us to adopt an alternative analysis strategy than in other studies of self-prioritization. The standard way to investigate SPE is to separately analyze matching and non-matching trials. This strategy, however, introduces serious problems if one intends to look independently at unbiased influences of a cue and a target (see [20] for a relevant discussion). First, it means that in the matching trials the influence of the cue and a target is confounded: matching trials are by definition the trials in which both stimuli are associated with the same identity, so it is impossible to determine whether any observed effect is caused by one or the other. At the same time, the mismatching trials are biased in a way that if, for example, we are interested in estimating the influence of a self-associated target on reaction times, then we need to take into account that in the non-matching trials by definition a self-associated target will never be accompanied be a self-associated cue. And if association of a cue influences reaction times (and it does, as shown by [20, 43]), then any effect that we observe in such situation may be explained by such imbalance of associations of accompanying cues. These concerns make the standard method of analyzing results of a matching task inadequate for studies which intend to independently investigate the influence of cues and targets. Therefore, in our analyses we adopted an alternative approach and decided not to focus on the distinction between the matching and mismatching trials (although we include the results of traditional analyses in the S1 Text), but instead to treat the identity of a cue and the identity of a target as two main factors in the analysis. Such approach would be problematic with the standard version of the task which is burdened with the fact that different numbers of trials are used to estimate different conditions. It was not an issue with our design due to the fact that after every cue there was an equal probability of seeing each target, and vice versa. However, our design and analysis strategy comes with two caveats, which need to be taken into account when interpreting the results. First, it does not allow us to directly look at the differences between patterns of results for matching and non-matching trials but only indirectly, by investigating the pattern of results reflected by the interaction effect. Second, it means that participants are biased to respond that a seen pairing was non-matching. However, in our design this bias was equal for each association of a cue, and therefore was orthogonal to our main comparisons of interest.

### Methods

#### Participants

Power analysis was performed with G*Power 3 software [54] in order to estimate the required sample size (the same power analysis applies to all three experiments). The analysis was conducted for one-way repeated measures ANOVA with three within-subject measurements for estimated effect size *f* = 0.4, α = 0.05, β = 0.95, assumed correlation among variables = 0.5, and non-sphericity correction ε = 0.8, yielding suggested sample size of 21 participants. In order to account for all possible combinations of associations the target sample size was chosen to be 24 participants.

Twenty-four people participated in the study, aged between 18 and 41 years (*M* = 21.9, *SD* = 4.39); half were women. Three participants were left-handed and one was ambidextrous. The participants represented diverse cultural backgrounds. All participants were fluid in English, but only fourteen were native English speakers, while others spoke Asian or European languages. Because faces used for each participant were matched to ethnicity, 17 participants performed the task with Asian faces and 7 participants with White faces. All participants had normal or corrected-to-normal vision. Informed consent was obtained from all participants before the start of the experiment according to procedures approved by the Monash University Human Research Ethics Committee (MUHREC; applies to all described experiments).

#### Procedure

**Fig 1:**
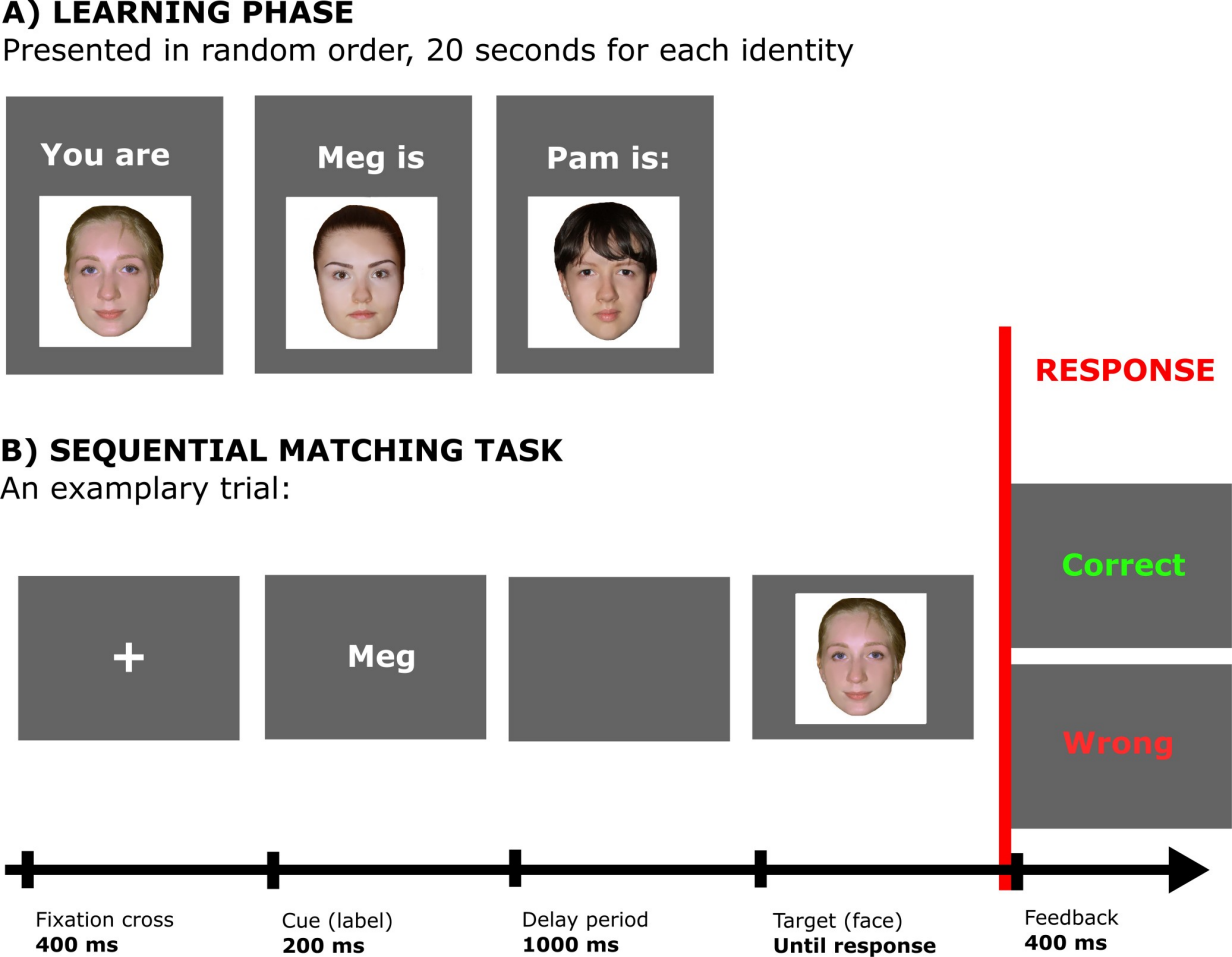
The procedure of Experiment 1. (A) During the learning phase participants were told which face corresponds to which identity, as indicated by a label “You”, and two names. (B) In the matching task participants were first presented with a label and then, after a 1 second delay, with a face. Their task was to judge whether the face matched with the label or not. Feedback was provided immediately after providing the response. (The faces in Fig 1 are for illustrative purposes only and they are not the actual faces used in the experiment; the individuals in the figure gave written consent to publish their photographs).

The experiment was programmed and conducted using Matlab R2013a with Psychophysics Toolbox (version 3.0.10; experimental script for this and other experiments, as well as the results can be freely accessed under the following link: https://osf.io/2q9w7). The experimental procedure consisted of two stages: learning phase and matching task. During the learning phase participants were presented with three pairings of a label and a face (Fig 1). Pairings were displayed on a computer screen in random order for 20 seconds each. This timing was chosen to match other previous studies of self-prioritization effect. All faces were taken from a database of faces [55] and were unfamiliar to participants. One of these faces was then associated with the participant through the pronoun “You”, and two other faces were associated with strangers by giving them names of other people (“Pam” and “Meg” for women, “Rob” and “Ned” for men). If any of the strangers’ names coincided with the participant’s real name, then this name was changed (to “Kat” for women and “Ken” for men). We used the pronoun “You” to indicate self-association, because the majority of previous studies on SPE used this word, and our pilot studies suggested that using a pronoun “I” does not change the pattern of results in experiments on self-prioritization.

The learning phase was followed by a matching task during which participants had to judge whether a label and a face displayed in a sequence are matching or not. The matching task consisted of a short practice block consisting of 24 trials, and three blocks with 90 trials each (amounting to 270 trials). The data obtained during these three blocks was used for analysis. Each trial started with a fixation cross presented for 400ms, which was followed with presentation of one of the names/labels for 200ms, and then with a delay period of 1 second. After the delay period, one of the faces was displayed and the participant’s task was to judge whether the face matched with the label by pressing a key on a keyboard indicating either a match or a mismatch (using “z” and “m”, key assignment was counterbalanced across participants). Feedback was provided immediately following the keypress, and displayed for 400ms. If no response was registered within 2000ms then participants were presented with a “No response” information for 400ms. The inter-trial interval was 600ms. There was equal probability (33.3%) of the first stimulus being associated with each identity, and the same applies to the second stimulus conditioned on the first, i.e. for every label there was equal probability (33.3%) that it will be followed by each of the three faces. The order of the trials was randomized, and the assignment of faces to the identities (names/labels) was counterbalanced across participants.

#### Stimuli

The experiment was conducted on a PC with a 22-inch LED monitor. All written stimuli including the labels were white, presented on a grey background. All labels used in the matching task had the same length of three letters and therefore the same width of 2°. The fixation cross was 0.6°x0.6°. Pictures of the unfamiliar faces were taken from the Chicago Face Database [55]. The total size of the pictures was 8°x8°, and the size of the face alone was approximately 3.8°x5.3°. Three female and three male faces were chosen separately from among the Asian and White faces. The facial expression of all of the faces was neutral. The groups of faces were chosen to make them similarly different from each other. The pictures from the database were subjected to additional editing to remove the neck and upper part of the trunk from the picture and replace it with a homogenous white color background. The following faces were used in the study: Asian women (AF-218-157-N, AF-230-193-N, AF-209-006-N), Asian men (AM-239-147-N, AM-250-149-N, AM-251-124-N), White women (WF-209-052-N, WF-201-156-N, WF-233-112-N), White men (WM-003-002-N, WM-015-002-N, WM-022-001-N).

#### Design, data processing and analysis

The experiment comprised a two-way repeated measures 3x3 design with the first factor being identity associated with a cue (self vs. stranger 1 vs. stranger 2), and the second factor being identity associated with a target (self vs. stranger 1 vs. stranger 2). The dependent variables were average reaction times and error rates. Mauchly's test for each ANOVA was conducted to check if the assumption of sphericity was violated, and Greenhouse-Geisser correction was applied where this violation was found. If the main effect of ANOVA was significant, planned contrasts using an orthogonal Helmert contrast were conducted in order to assess statistical significance of the differences between conditions: the first contrast was between the self and an average of Stranger 1 and Stranger 2, and the second contrast was between Stranger 1 and Stranger 2.

Data processing and analysis were conducted using custom scripts written in MATLAB with Statistics and Machine Learning Toolbox. Bayesian analyses were conducted using JASP 0.9.0.1. Only trials with correct responses were included in the analysis of reaction times. Correct trials with RTs shorter than 200ms were removed. Moreover, correct trials with RTs longer than 2.5 median absolute deviations (MAD) over the median for each participant were excluded, as suggested by [56]. This procedure led to exclusion of on average 7.0% (*SD* = 5.5%) of trials in Experiment 1.

### Results

**Fig 2:**
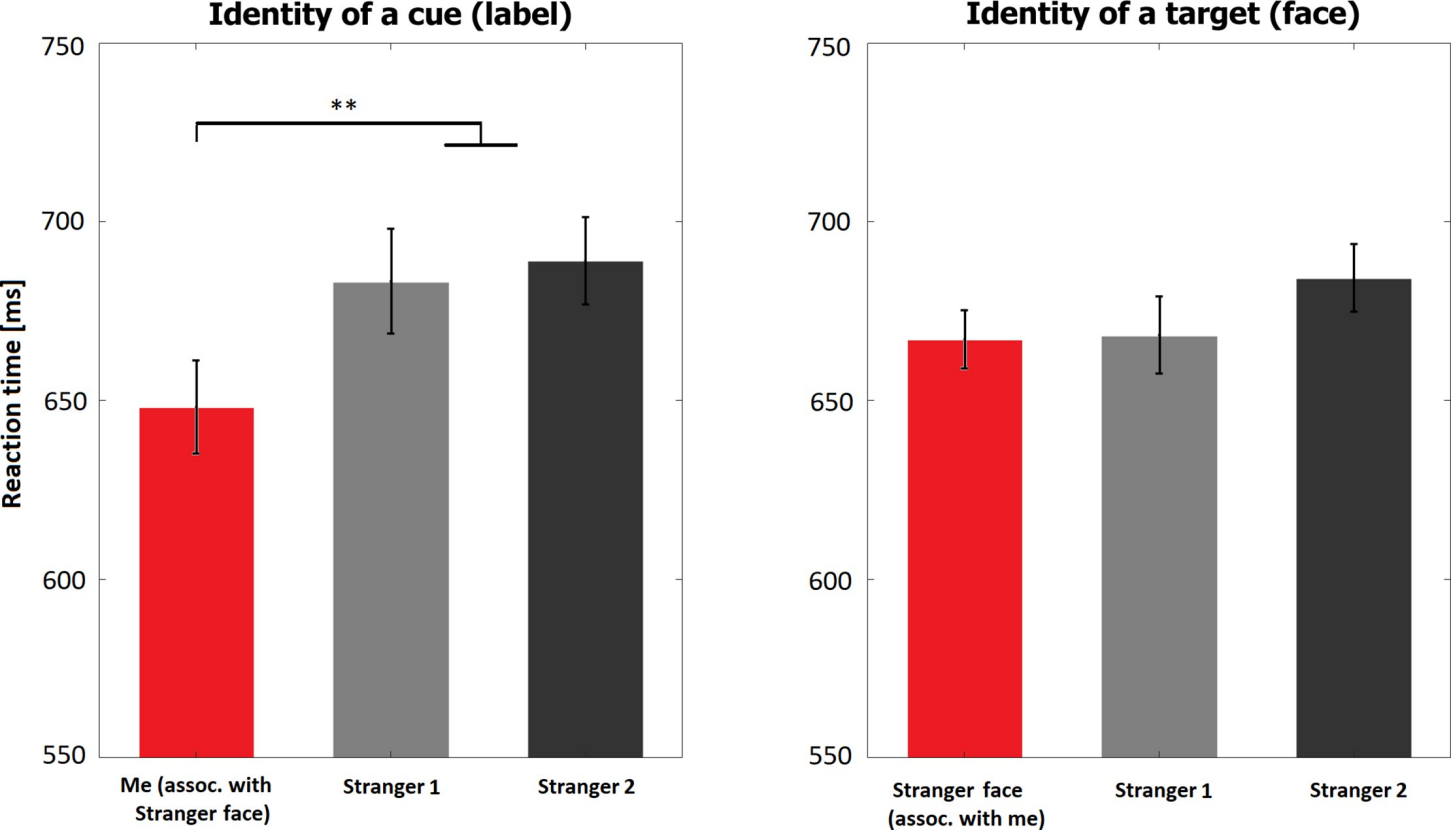
Results of Experiment 1. Red bar represents average reaction time for self-related condition, and grey bars for stranger-related conditions. * reflects statistical significance below 0.05, and ** below 0.01.

We conducted a two-way repeated-measures 3x3 ANOVA on reaction times to investigate the influence of identity of the label presented as a cue, and of the face presented as a target. For the influence of a cue, the main effect of identity was significant (*F*(2, 46) = 7.48, *p* = 0.002, partial η^2^ = 0.25). Planned contrasts showed that in trials beginning with the word “You” participants were significantly faster than in trials beginning with strangers’ names (*p*<0.001, BF_10_ = 37.2; see Fig 2, Table 1 presents all descriptive statistics). The difference between the two stranger-identities was not significant (*p* = 0.64, BF_10_ = 0.24). The association of the target, a face, had no effect on reaction times (*F*(2, 46) = 2.56, *p* = 0.09, partial η^2^ = 0.10). The interaction effect between label and face was also significant (*F*(4, 92) = 11.33, *p*<0.001 Greenhouse-Geisser corrected, partial η^2^ = 0.33), reflecting the fact that participants were faster in the matching than mismatching trials (see S2 Text for figures illustrating all interaction effects).

**Table 1:** Mean reaction times and accuracy (standard deviation in brackets) for all three experiments.

	Cue (label)			Target (face)		
**EXPERIMENT 1**	**Me (assoc. with stranger face)**	**Stranger 1**	**Stranger 2**	**Stranger face (assoc. with me)**	**Stranger 1**	**Stranger 2**
**Reaction Time Accuracy**	648.2 (126)	683.3 (118)	689.1 (131)	667.1 (121)	668.3 (118)	684.3 (126)
	0.936 (0.06)	0.931 (0.06)	0.922 (0.07)	0.941 (0.05)	0.930 (0.05)	0.918 (0.08)
**EXPERIMENT 2**	**Stranger 1**	**Stranger 2**	**Stranger name (assoc. with my face)**	**Stranger 1**	**Stranger 2**	**My face (assoc. with a stranger name)**
**Reaction Time Accuracy**	655.6 (108)	661.7 (101)	628.6 (97)	660.1 (94)	663.8 (113)	621.0 (96)
	0.921 (0.08)	0.913 (0.07)	0.920 (0.07)	0.907 (0.08)	0.917 (0.06)	0.930 (0.06)
**EXPERIMENT 3**	**Me (assoc. with Stranger 1 face)**	**Stranger 2**	**Stranger 3 name (assoc. with my face)**	**Stranger 1 face (assoc. with me)**	**Stranger 2**	**My face (assoc. with Stranger 3 name)**
**Reaction Time Accuracy**	635.8 (102)	636.1 (115)	642.6 (111)	651.9 (110)	640.7 (104)	620.6 (104)
	0.949 (0.04)	0.962 (0.04)	0.944 (0.03)	0.954 (0.03)	0.958 (0.04)	0.943 (0.03)

There was no effect of identity of the label (*F*(2, 46) = 2.31, *p* = 0.11, partial η^2^ = 0.09) nor face (*F*(2, 46) = 2.12, *p* = 0.13, partial η^2^ = 0.08) on accuracy. However, the interaction between the label and the face was significant (*F*(4, 92) = 4.12, *p* = 0.004, partial η^2^ = 0.15) reflecting the fact that participants were less accurate in matching than in mismatching trials.

### Discussion

Results of the first experiment replicated previous findings and did not find evidence for modulation of bottom-up processing of faces by arbitrary self-association. This suggests that faster detection of one’s own real face, which has been frequently reported in the literature, may be due to increased familiarity and not just mere self-association (but see: [57]).

Moreover, our study shows that the self-prioritization effect in a sequential task cannot be fully explained by perceptual expectations induced by the cue in a situation in which the self-associated cue informed participants that there is an increased probability that the target will also be self-related. As such, our results might support the interpretation of the top-down effect from [43] (but also [58–60]), as being caused by attentional mechanisms, although other potential mechanisms (cognitive, motor) can be also involved. At the same time, our results do not rule out the possibility that perceptual expectations alone can lead to similar outcomes. However, testing this would require designing a different experimental paradigm.

## Experiment 2

In Experiment 2 we reversed the idea behind Experiment 1, and rather than associating a stranger’s face with the self, we associated one’s own face with a stranger’s name. As such, we were able to test whether real faces lead to a bottom-up self-prioritization effect. We reasoned that if we observe faster reaction times for one’s real face presented as a target (irrespective of the cue), then it will suggest that arbitrary self-association of a stranger’s face does not modulate bottom-up mechanisms, while self-association of one’s real face does it, leading to the conclusion that these two types of stimuli are prioritized using different mechanisms. Conversely, if we fail to observe bottom-up prioritization of participants’ real faces it may indicate that real faces are prioritized using the same mechanism as self-associated faces.

Moreover, by replacing the label “You” with an arbitrary name, we were able to control for an alternative explanation of the top-down effect in Experiment 1, i.e. that the effect was caused by grammatical distinctiveness of the label “You”, which was a pronoun among two proper nouns [61], rather than by its self-association (for an example of a similar control see Experiment 1 in [43]). We expected that associating a stranger’s name with one’s real face will lead the participants to represent this name as self-related and, consequently, that presentation of this name as a cue will lead to a top-down self-prioritization effect.

### Methods

#### Participants

Twenty-nine people participated in Experiment 2. Five participants were excluded from the analysis due to technical issues during the preparation of photographs. The age of the remaining 24 participants ranged between 18 and 35 years (*M* = 21.8, *SD* = 3.65). Half of them were women. Three participants were left-handed. Seventeen participants performed the task with Asian faces and seven participants with White faces. Thirteen participants were native English speakers. All participants had normal or corrected-to-normal vision.

#### Procedure and stimuli

The procedure was identical to Experiment 1, except for two changes. First, there was no identity-label representing the participant and therefore the label “You” was not used in the task. Instead, each participant was presented with three names of the same gender: ‘Liz’, **‘**Meg’, **‘**Pam’ for women, and ‘Rob’, **‘**Ned’, **‘**Sam’ for men. If the participant’s name coincided with one of them then the relevant name was changed to **‘**Kat’ for women or **‘**Ken’ for men. Second, one of the pictures was replaced with a photograph of the participant him- or herself. The two other faces were taken from the Chicago Faces Database [55] as in Experiment 1. We used the following faces: Asian women AF-218-157-N, AF-230-193-N, Asian men AM-239-147-N, AM-250-149-N, White women WF-209-052-N, WF-201-156-N, White men WM-003-002-N, WM-015-002-N.

The photographs of participants were taken with a high-quality digital photo camera at the beginning of each experiment. To ensure maximal similarity to the faces from the Chicago Faces Database the participants were seated in a controlled environment on a chair in front of the camera with white background. They were illuminated by two lamps and a detachable camera flashlight. The participants were told to remove glasses if they wore them, and to pull their hair back if they had long hair, in order to make their hairstyle similar to that of selected people from the faces database. The photographs were then subjected to the same treatment as faces from the database in Experiment 1, i.e. the background as well as the neck and the trunk were cut from the picture and replaced with a homogenous white color background and contrast/brightness/hue corrections were applied. The pictures were not mirror-reversed in order to make them more unfamiliar to participants, and therefore make the images of participants’ faces more comparable to other pictures in regard to visual familiarity.

#### Design and data analysis

The experimental design and planned analyses in Experiment 2 were the same as in Experiment 1: we conducted a two-way 3x3 ANOVA for identity of a cue and a target, which was followed with planned Helmert contrasts (contrast 1: self vs average strangers; contrast 2: stranger 1 vs. stranger 2) if the main effect of association of a label or a face was significant.

### Results

**Fig 3:**
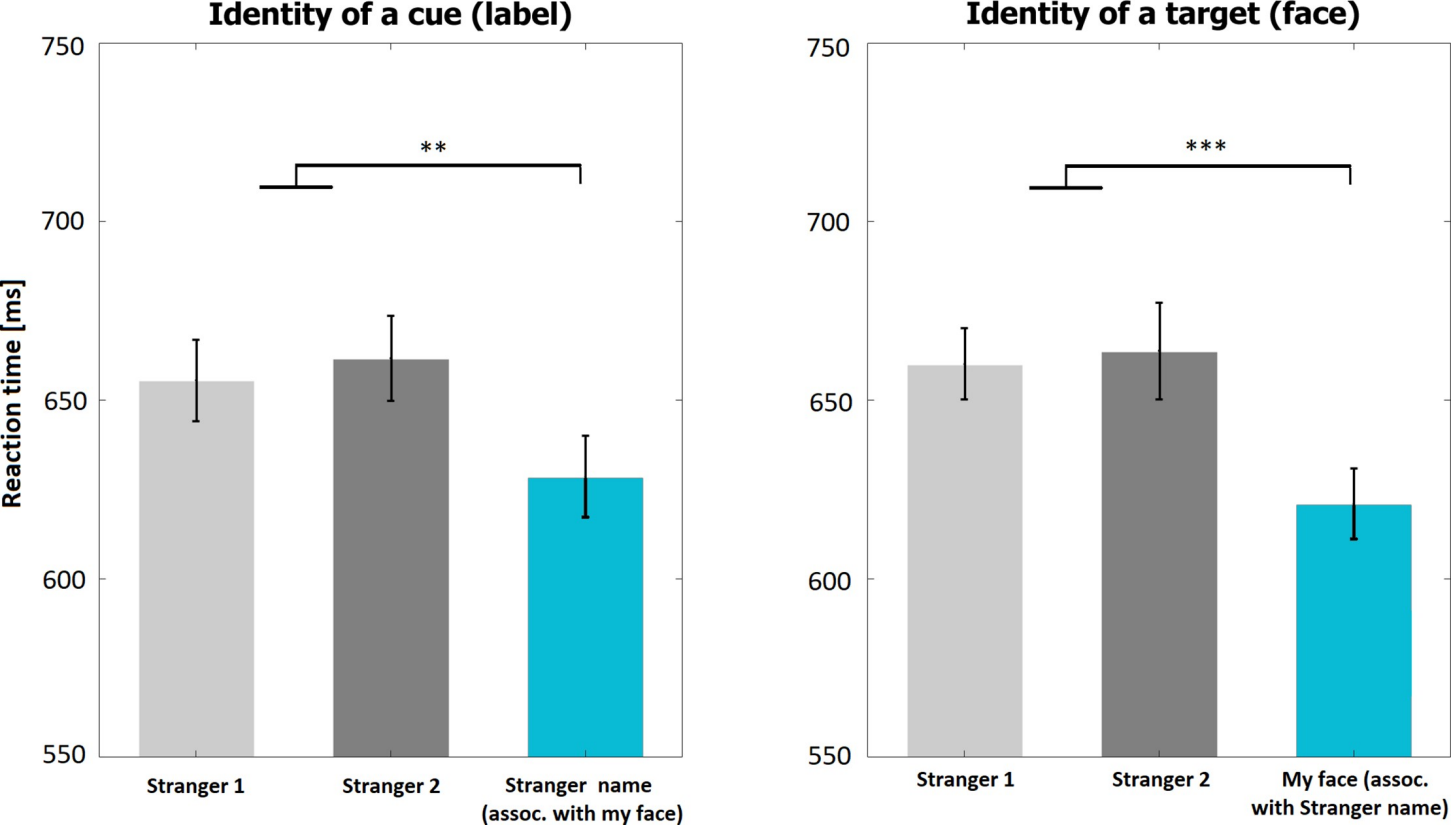
Results of Experiment 2. Blue bar represents average reaction time for condition with participant’s real face which has been associated with a stranger’s identity, and grey bars represent stranger-related conditions. * reflects statistical significance below 0.05, ** below 0.01, and *** below 0.001.

In the second experiment 8.2% of trials (*SD* = 6.3%) were removed as outliers or due to incorrect responses. The main effect of association of a cue (name) on reaction times was statistically significant (*F*(2, 46) = 6.78, *p* = 0.003, partial η^2^ = 0.23). If a cue was a stranger’s name which had been associated with a participant’s real face, then the reaction times were faster than for other names (*p* = 0.002, BF_10_ = 17.4). The difference between reaction times following stranger_1 and stranger_2 names was not significant (*p* = 0.56, BF_10_ = 0.25). The main effect of identity of the face on reaction times was also significant (*F*(2, 46) = 11.75, *p*<0.001 Greenhouse-Geisser corrected, partial η^2^ = 0.34). Reaction times were shorter in trials in which target was a participant’s own face than when it was a face of a stranger (*p*<0.001, BF_10_ = 1377.6), while the difference between two strangers’ faces was not significant (*p* = 0.74, BF_10_ = 0.23). Fig 3 illustrates the results. The interaction effect was also significant (*F*(4, 92) = 4.36, *p* = 0.003, partial η^2^ = 0.16) reflecting faster reaction times in matching trials (see: S2).

There was no effect of identity of the label (*F*(2, 46) = 0.27, *p* = 0.76, partial η^2^ = 0.01) nor face (*F*(2, 46) = 1.88, *p* = 0.16, partial η^2^ = 0.08) on accuracy, but their interaction was significant (*F*(4, 92) = 23.47, *p*<0.001 Greenhouse-Geisser corrected, partial η^2^ = 0.51), reflecting lower accuracy in matching than mismatching trials.

### Discussion

In Experiment 2 we found evidence of both top-down and bottom-up self-prioritization effects. First, we found a strong prioritization effect in reaction times for one’s own face, as compared to strangers’ faces. This shows that the lack of bottom-up effect for self-associated face in Experiment 1 cannot be attributed to specific characteristics of the sequential matching task, but rather to differences in mechanisms underlying prioritization of an arbitrary and real self-face. We will discuss this finding in a wider context in the general discussion, including to what extent it can be attributed to familiarity.

Second, we found evidence that associating one’s own face to a different person’s name causes that name to trigger the top-down self-prioritization effect, i.e. presentation of a stranger’s name that has been associated with one’s own face leads to faster processing of any ensuing face. This result also shows that the top-down effect detected in Experiment 1 cannot be explained by distinctiveness of the label “You”; if this was the case then we would not observe any effect in Experiment 2. Moreover, the effect size of identity of a cue in Experiment 2 was practically the same as in Experiment 1 (partial η^2^ = 0.21 and 0.23, respectively), suggesting that neither did grammatical distinctiveness play a role in the first experiment.

## Experiment 3

In Experiment 3 we tested whether the top-down self-prioritization effects observed in the previous two experiments can be attributed to two independent top-down cognitive processes or to the same one top-down process. In order to test this, we combined experiments 1 and 2 into one study in which participants were asked to simultaneously associate a stranger’s face with the self, and one’s own face with a stranger’s name.

Our reasoning was that if the two cue-induced top-down effects from the previous two experiments are independent effects, then we should observe both of them in Experiment 3. On the other hand, if they conflict in Experiment 3, leading to longer response times for both self-associated cues and consequently to either disappearance of the effect or even the reverse effect (fastest reaction times following cueing with a stranger’s name which has been associated with a stranger’s face) then it would provide a strong argument that they reflect the same underlying cognitive process (or at least that the underlying processes are highly interdependent). Moreover, if these two top-down effects reflect the same one process, we expected that the magnitude of each effect (calculated as the difference in RTs following cueing with each label minus RTs following the label reflecting a stranger with a stranger’s face) will be positively correlated. Conversely, if they reflect two distinct processes, we expected no correlation. Additionally, we expected to replicate the findings from Experiments 1 and 2 regarding the bottom-up effect of the target, i.e. we expected faster reaction times after presentation of one’s real face, and no such effect after presentation of a self-associated stranger’s face, irrespective of the preceding cue.

### Methods

#### Participants

Twenty-five people participated in Experiment 3. One person was excluded from the analysis due to technical issues with the stimuli. The age of the remaining 24 participants ranged between 19 and 39 years (*M* = 24.7, *SD* = 5.86). Half of them were women. Two participants were left-handed. Twelve participants performed the task with Asian faces and twelve with White faces. Ten participants were native English speakers. All participants had normal or corrected-to-normal vision.

#### Procedure and stimuli

The procedure was the same as in Experiment 2, with one difference. Instead of three names, the cues consisted of two names and one label “You”. The label “You” was always associated with one of the faces from the database, while the picture of a participant’s real face was always associated with one of the names. The names used in the study were the same as in Experiment 1. In cases when the participant’s real name coincided with one of the names from the experiment, the same procedure was applied as in Experiment 1.

#### Design and data analysis

The experimental design in Experiment 3 was the same as in Experiments 1 and 2: we conducted a two-way 3x3 ANOVA for identity of a cue and a target. However, if the main effect of a cue or a target was significant we followed it with Tukey’s HSD post hoc tests, because we were interested in the full pattern of differences.

### Results

**Fig 4:**
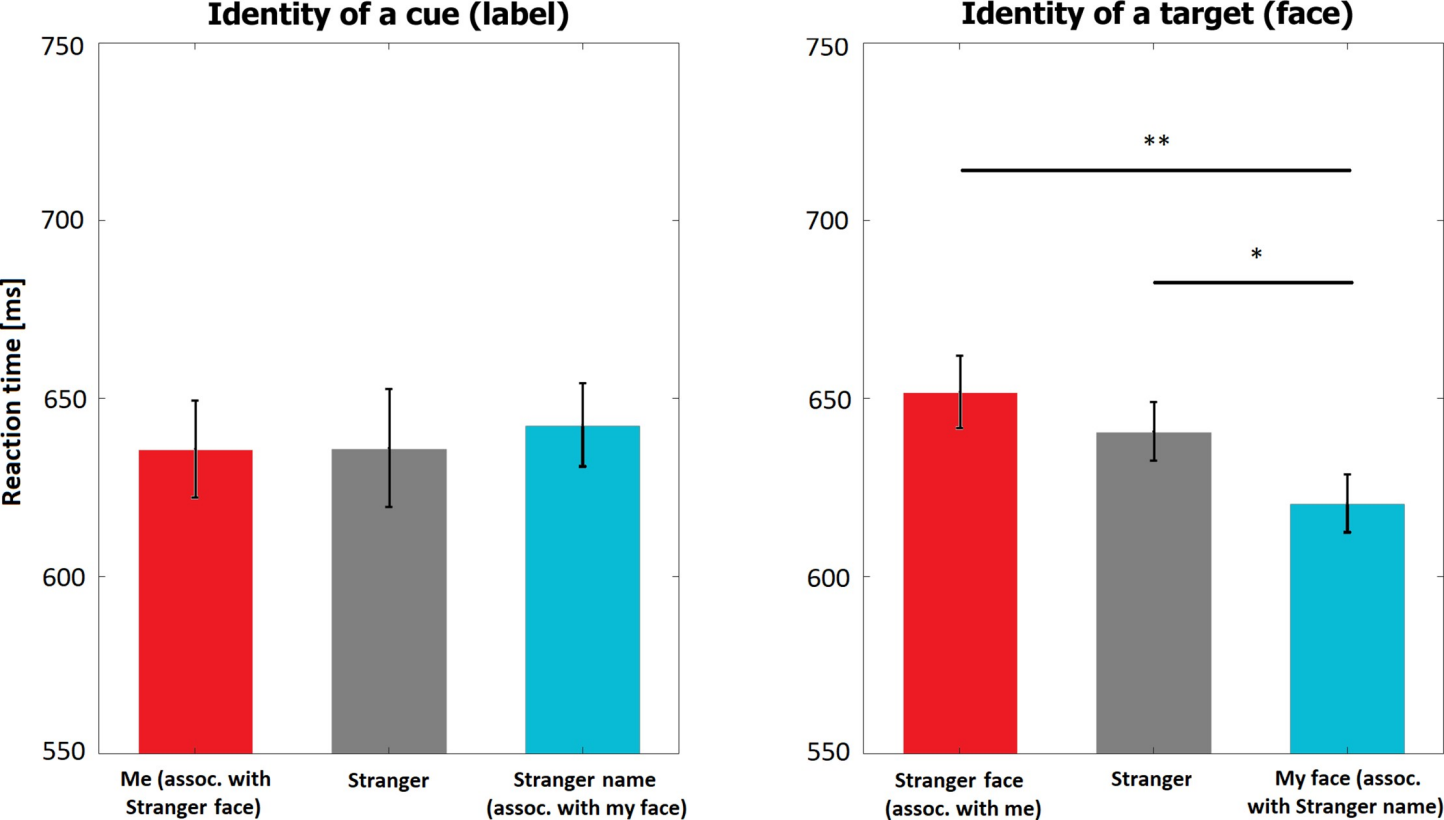
Results of Experiment 3. Red bars represent the same manipulation as in Experiment 1 (stranger’s face associated with the participant), blue bars represent the same manipulation as in Experiment 2 (participant’s real face associated with stranger’s identity), and grey bars represent fully stranger-related condition. ** reflects statistical significance below 0.01, * below 0.05.

On average 4.9% (*SD* = 2.8%) of the trials were excluded due to outliers or incorrect responses. One-way repeated measures ANOVA detected no difference in the influence of the identity of the cue on reaction times (*F*(2, 46) = 0.19, *p* = 0.83, partial η^2^ = 0.01). ANOVA conducted in order to assess the influence of the identity of a target (face) detected a significant main effect (*F*(2, 46) = 7.7, *p* = 0.001, partial η^2^ = 0.25). Reaction times for one’s real face were faster than for a stranger’s face (*p* = 0.028, BF_10_ = 8.13) and a self-associated stranger’s face (*p* = 0.006, BF_10_ = 30.80). The difference between the latter two was not significant (*p* = 0.8, BF_10_ = 0.27). Fig 4 illustrates the results. The interaction effect between these two factors was also significant (*F*(4, 92) = 8.98, *p*<0.001, partial η^2^ = 0.28) reflecting faster reaction times in matching than mismatching trials.

In order to assess the relationship between the effects caused by the cue referring to a self-associated stranger’s face (label “You”) and the cue referring to a stranger-associated own real face (e.g., label “Meg”), we performed an additional correlational analysis. We calculated Spearman’s ranked correlation between measures of the top-down self-prioritization effects caused by these cues. Spearman’s correlation was used because of a relatively small sample size. The baseline condition were trials in which the cue was a stranger’s name associated with a stranger’s face (e.g., “Liz”). The prioritization effect caused by the label “You” was calculated as the difference between average RTs following presentation of the label “You”, and average RTs following the baseline: name of a stranger with a stranger’s face (e.g. RT_Label: “You”_—RT_Label:”Liz”_). The prioritization effect caused by the name associated with one’s real face was calculated in an analogous way: as the difference between average RTs following the name representing a stranger with one’s real face, and average RTs following the baseline: name of a stranger with a stranger’s face (e.g. RT_Label:”Meg”_—RT_Label:”Liz”_). The correlation between these two effects was strong and significant (*rho* = 0.66, *p*<0.001).

There was no effect of identity of the cue (*F*(2, 46) = 1.98, *p* = 0.15, partial η^2^ = 0.08) nor target (*F*(2, 46) = 2.81, *p* = 0.07, partial η^2^ = 0.11) on accuracy, but their interaction was significant (*F*(4, 92) = 11.62, *p*<0.001, partial η^2^ = 0.34), what was driven by worse accuracy in matching than mismatching trials.

### Discussion

Results of the third experiment suggest that the top-down effects caused by the cue found in Experiment 1 (the label “You”) and Experiment 2 (the name associated with one’s real face) might be two manifestations of the same underlying cognitive process. If they were fully independent then they would both manifest themselves in a task in which both of them are elicited, and their strength would be comparable to the strength observed in tasks in which they appear individually (this observation also applies to potential criticism that any effect caused by presentation of the label “You” is due to its grammatical distinctiveness, because the magnitude of facilitation by grammatical distinctiveness should not be related to the magnitude of effects caused by self-association). Results of Experiment 3 show that this was not the case. Moreover, the fact that we have not observed the effect could not be due to lack of experimental power, because RTs following cueing with a name associated with one’s real face were even slower than to a stranger’s name associated with a stranger face, and RTs following the label “You” were virtually identical with the latter. Second, the magnitude of the self-effect for both self-related conditions correlated with each other, providing a positive argument for their interdependence. Hence, we argue that the top-down self-prioritization effects from Experiments 1 and 2, might be in fact two manifestations of the same process. It is also possible that they don’t reflect one, but two distinct, although interdependent, top-down processes. While we cannot decisively rule out such possibility, we believe that in that case, we would observe a more complex pattern of results, such as a decrease of magnitude of both effects, or preservation of only one of them.

Reaction times following presentation of the target corroborated findings from the two previous experiments regarding the bottom-up mechanisms of self-prioritization, i.e., people responded faster (irrespective of what was the cue) if the target was a picture of their real face, but not when it was a self-associated stranger’s face.

## General discussion

Our study attempted to reveal and disentangle two independent cognitive mechanisms driving prioritization of self-related stimuli. Across three experiments, we investigated top-down (cue-induced) and bottom-up (target-induced) effects of both established and arbitrary self-associations by comparing processing of participants’ real faces and self-associated strangers’ faces. In Experiment 1 we replicated the finding from [43] showing that self-association of a cue in a sequential match-non-match task leads to facilitated processing of an ensuing target irrespective of whether a target is self-associated. Importantly, we found that this top-down self-prioritization effect cannot be explained by the fact that a self-associated cue increases perceptual expectation that the target will be associated with the self, because in our version of the task, cues did not convey any predictive information about the identity of the target (there was an equal probability that the target would be related to each of three identities). This suggests that this top-down effect does not need mechanisms responsible for perceptual expectation to emerge (see: [46]). This insight might provide empirical support for the claim made in current literature that self-related effects are driven by modulation of attention [58–60, 62]. However, two recent studies have provided experimental evidence suggesting that self-prioritization affects not perceptual, but cognitive (e.g. decision making) [34] and motor stages of processing [26] (but see [19, 63] for evidence of modulation of perception). These studies are relevant, because top-down attention is usually regarded as affecting perception, rather than decision making or motor response. Hence, the top-down effect demonstrated in our study may reflect facilitation of perception, decision making, response, or any combination of them. Unfortunately, our results do not allow us to directly address this issue. It is worth adding that it is possible that even though perceptual expectations are not necessary to induce top-down self-prioritization, they may be sufficient to induce it. Future studies should try to elicit this effect through manipulation of perceptual expectations alone.

The goal of the second experiment was to investigate bottom-up and top-down processing in a task in which one’s real face was associated with a stranger’s name. We found that a) the picture of one’s real face displayed as a target is processed faster even after associating it with a stranger’s name, and b) a similar top-down effect (generalized facilitation of RTs caused by the cue) to the one observed in Experiment 1 can be elicited by showing a name arbitrarily associated with one’s real face, irrespective of which target occurs. When contrasted with the results of Experiment 1, these results show, first, that faster reaction times following presentation of one’s real face cannot be due to only self-association, but have to be caused by some additional factor (which stands in partial opposition to the results from previous self-prioritization paradigms). Second, the fact that there was a top-down effect in Experiment 1 cannot be fully explained by distinctiveness of the label “You [61], because if that was the case then there would be no top-down effect in Experiment 2.

The results of Experiment 3 provide evidence supporting the claim that the top-down self-prioritization effects from the first two experiments reflect the same one underlying cognitive mechanism. This is shown by the fact that, first, the effect vanishes when both associations are present in one task, presumably because they interfere with each other. Second, the magnitude of both effects is strongly correlated across participants indicating substantial statistical interdependence.

Across three experiments we have reliably found that participants responded faster when the target stimulus was their own face (in Experiments 2 and 3), and we haven’t found such an effect when the target was a self-associated stranger’s face (in Experiments 1 and 3). In all cases, these effects on reaction times were independent of the identity of a cue, and therefore they reflect bottom-up (cue-independent) processing. These results strongly suggest that the classical, well-established finding that people are faster when recognizing their own face rather than other faces cannot be explained by self-association alone, because if this was the only driving factor then we would observe bottom-up prioritization of arbitrary self-associated faces as well as prioritization of participants’ real faces. An obvious factor explaining our pattern of results is *familiarity*. While a stranger’s face was novel to participants, their own face was well known to them. Given that it is well-established that familiar information is processed faster and more accurately than unfamiliar information (e.g. [2, 64–66]) our results may suggest that the sole factor responsible for the difference is familiarity. However, previous research on perception of one’s face found evidence of self-prioritization even when contrasted with highly familiar faces of close others and famous individuals [3, 5, 7, 57, 67]. This suggests that self-related effects may transpire not only from familiarity, but also from the interaction between self-association and the process of learning. It is possible that self-related content of memory enjoys preferential treatment through deeper encoding and consolidation of memory traces than other-associated information (even if exposure is objectively identical), a notion that resonates with the “self as an integrative glue” proposal [59] and is supported by research on autobiographical memory and self-reference effects ([10, 16–18, 68], also: [24], cf. [31]). Moreover, results of a recent study on self-prioritization effect [32], in which decreasing the frequency of presentation of other-related, but not self-related, arbitrary pairings led to decrease of detection speed and accuracy, can be seen as further support for this hypothesis.

Even though neither the present study, nor [43] have found evidence of bottom-up prioritization of arbitrary self-associated faces in reaction times, Woźniak et al. [43] reported EEG evidence to the effect that presentation of a self-associated stranger’s face led to decreased amplitude of the frontal N2 event-related component, regardless of whether a self-associated face was presented as a cue or a target. They found this effect in two independent samples, drawing attention to the fact that the same effect on the frontal N2 has been observed when people perceive their real faces [69, 70]. These results suggest that perhaps also in our experiments, the difference between real participants’ faces and the self-associated ones cannot be exclusively attributed to familiarity.

Our study complements the growing field of research on cognitive mechanisms underlying and affected by self-prioritization. While our focus was on dissociation of top-down and bottom-up mechanisms, other studies focused on different aspects of self-prioritization. For example, several recent studies have investigated whether arbitrary self-association affects processes related to spatial attention in a visual search task [36, 71] and in a spatial cuing tasks [21, 33], finding evidence that only in specific contexts can such modulation take place. Other studies have investigated whether self-associated stimuli have privileged access to consciousness, but have found inconsistent results ([72] vs [35, 73]). Finally, yet further studies, discussed above, focused on determining whether self-prioritization affects perception, cognition, or motor responses [26, 34]. While our results do not speak directly to any of these issues, it may be beneficial to take into account the distinction between bottom-up and top-down processes in future research on these topics, for example by comparing responses to familiar self-associated stimuli with responses to stimuli for which self-association have been arbitrarily established.

More generally, our study suggests new ways to investigate neurocognitive mechanisms underlying self-related processing. The investigation of self-related modulation of top-down and bottom-up processes may prove especially relevant in the context of a recent trend to explain the self from the perspective of predictive processing and the free energy principle, which fundamentally operates with different kinds of modulation of top-down and bottom-up neuronal messaging (e.g. [74–83]). The predictive processing framework postulates that the brain operates as a prediction-machine based on the interplay between precision-weighted prediction errors and the hierarchical structure of generative models instantiating predictions. Sequential versions of the matching task used in this study allow dissociation of the phase during which one forms predictions (delay period following presentation of a cue) from the phase during which the brain compares predictions with sensory input (immediately following a target). As such, the sequential matching task helps to dissociate fundamental building blocks of the predictive processing model, and suggests ways to investigate to what extent self-association affects each of them. Moreover, the predictive processing account provides a theoretical grounding for the proposal that self-representation acts as an integrative hub or an “integrative glue” [59]. In this framework, the self-related effects can be interpreted as manifestations of differential processes of acquisition and updating of generative models (which are responsible for formulating predictions) for self-related and non-self-related hidden causes. One possibility is that internal models responsible for inferring self-related causes are characterized by a stronger learning rate than other models [84]. As a result, learning new associations with the self is much faster (reflecting initial greater malleability) than learning other associations, but as a direct consequence of this process these associations become stable and resistant to change (i.e., the learning rate decreases) quicker than other associations. Future research can test predictions of this model using computational models of learning in a predictive coding architecture (e.g. [85, 86]).

To conclude, our results suggest that there are at least two distinct types of the self-prioritization effect, underpinned by modulation of top-down and bottom-up processing respectively. The first one, in line with [43], reflects cue-induced activation of an abstract self-concept, which causes facilitated processing of the subsequent target through top-down mechanisms. The second type of prioritization, as illustrated by faster reaction times to one’s real face than to a self-associated stranger’s face, may be either due to increased familiarity of standard self-related stimuli (such as one’s face or name), or due to a different learning trajectory of self-related information (e.g. through stronger consolidation of self-related memory traces). Future studies should determine whether these are the only factors contributing to the effect, and what their exact underlying neural and cognitive mechanisms might be. In a wider perspective, our results provide a call to take into consideration the difference between top-down and bottom-up processing also in different experimental paradigms investigating self-prioritizaton [26, 34, 62, 63, 72, 87, 88].

## Supporting information

S1 Text A supplementary analysis investigating interaction between trial type (matching vs. mismatching) and identity. (DOCX)

S2 Text Supplementary figures illustrating Cue X target interaction effects. (DOCX)
