# Supplementary material for: Stranger to my face: Top-down and bottom-up effects underlying prioritization of images of one’s face
Source: PLoS One. 2020 Jul 9;15(7):e0235627. doi: 10.1371/journal.pone.0235627 (PMC7347180; doi:10.1371/journal.pone.0235627)
Supplement: S1 Text — (DOCX) [file pone.0235627.s001.docx]

**S1 Text. A supplementary analysis of interactions between trial type (matching vs. mismatching) and identity.**

The supplementary analyses were performed in order to further investigate the interaction between identity (e.g. self, stranger 1, stranger 2) and a factor left out of the analysis in the main text, namely of trial type, that is, whether the trials were matching or mismatching. For each experiment we conducted two 2-way ANOVAs with a 2x3 design. These two ANOVAs differed from each other in regard to the way in which non-matching trials were calculated, according to the procedure described in Woźniak, Kourtis, & Knoblich (2018). The procedure was as follows (let us assume that there were three identities in the matching task: self, Liz, and Meg). The first way to calculate the averages for non-matching trials for a given identity (for example for the self) is to compute means for trials in which **the cue** is associated with that identity (a label “You”) and the target is mismatching (a face of Liz or a face of Meg). This method of calculating non-matching trials allows one to look at the influence of the cue on reaction times. Following Woźniak et al., we will call averages obtained using this method “non-matching 1” or NM1. The second way to compute average RTs for the mismatching trials for a given identity (e.g. the self) is to take trials in which **the target** is associated with that identity (self-associated face in experiment 1), and the cue is mismatching in regard to the target (a label “Liz” or “Meg”). This way of calculating averages allows to look at the influence of identity of the target on RTs, and following Woźniak et al., will be called non-matching 2 or NM2.

In the remaining part of the supplementary material we report two analyses for each experiment: one contrasting matching trials with NM1-averaged mismatching trials, and one contrasting matching trials with NM2 trials. Because we were mainly interested in the interaction between trial type and identity, we report the results of the post hoc tests (using HSD test) only for (significant) interaction effects. Before proceeding to the results, we note one more thing - because the design of our study differed from the one employed by Woźniak et al., the averages in the matching trials were calculated using only half of the number of trials used to calculate non-matching averages. This does not make the results from the supplementary materials invalid, but makes averages of the matching trials more sensitive to random noise.

We report only the results of reaction times, because analyses of error rates yielded no significant results. A single exception was a significant main effect of NM1 trials in experiment 2: NM1: *F*(1,46)=1.46, *p*=0.24, partial *η^2^*=0.06, reflecting slightly higher accuracy in stranger_1-related (95.8%) and stranger_2-related trials (95.1%) than in self-related trials (94.5%). However, subsequent post hoc tests revealed that the difference was significant only between self and stranger_1 (*p*=0.047).

**Experiment 1.**

**Matching vs non-matching 1 (NM1)**

The analysis contrasting matching and NM1 trials in experiment 1 has shown significant main effects of both trial type (*F*(1,23)=21.72, *p*<0.001, partial *η^2^*=0.49), and identity (*F*(2,46)=7.50, *p*=0.002, partial *η^2^*=0.25). Their interaction was not significant (*F*(2,46)=1.67, *p*=0.2).


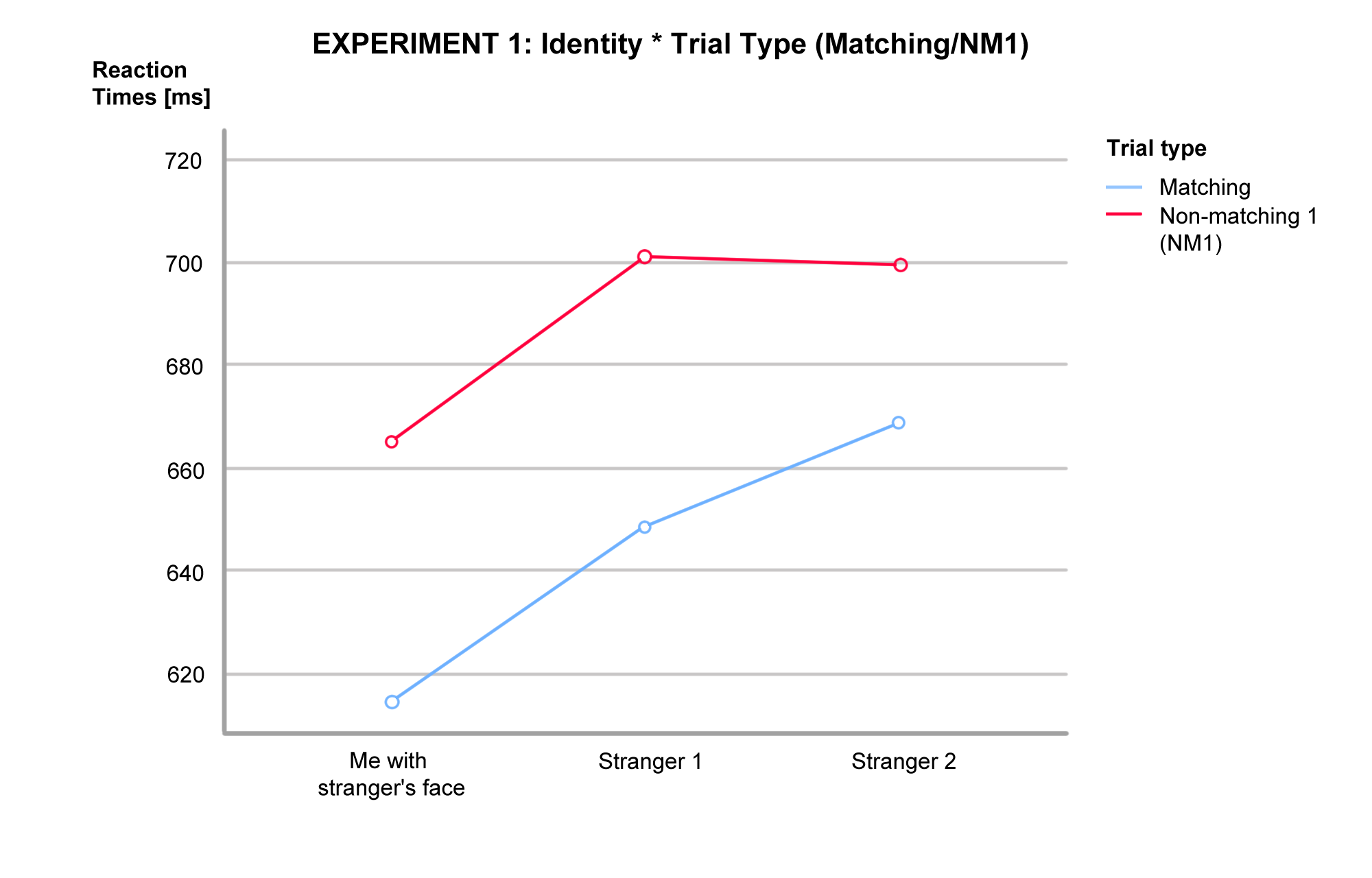


**Fig 1**. Average reaction times for matching and non-matching (NM1) trials for three identities (self-associated stranger’s face, stranger 1, stranger 2).

**Matching vs non-matching 2 (NM2)**

The analysis contrasting the matching trials with NM2 trials found significant main effects of trial type (*F*(1,23)=21.45, *p*<0.001, partial *η^2^*=0.48) and identity (*F*(2,46)=4.59, *p*=0.015, partial *η^2^*=0.17), but also significant interaction between them (*F*(2,46)=4.67, *p*=0.014, partial *η^2^*=0.17). Post hoc HSD tests of the interaction effect revealed that RTs in the matching trials for “Me with stranger’s face” were faster than RTs in all NM2 trials (all *p* values < 0.005) and faster than RTs for Stranger 2 in the matching trials (*p*=0.02). Other differences were not statistically significant.


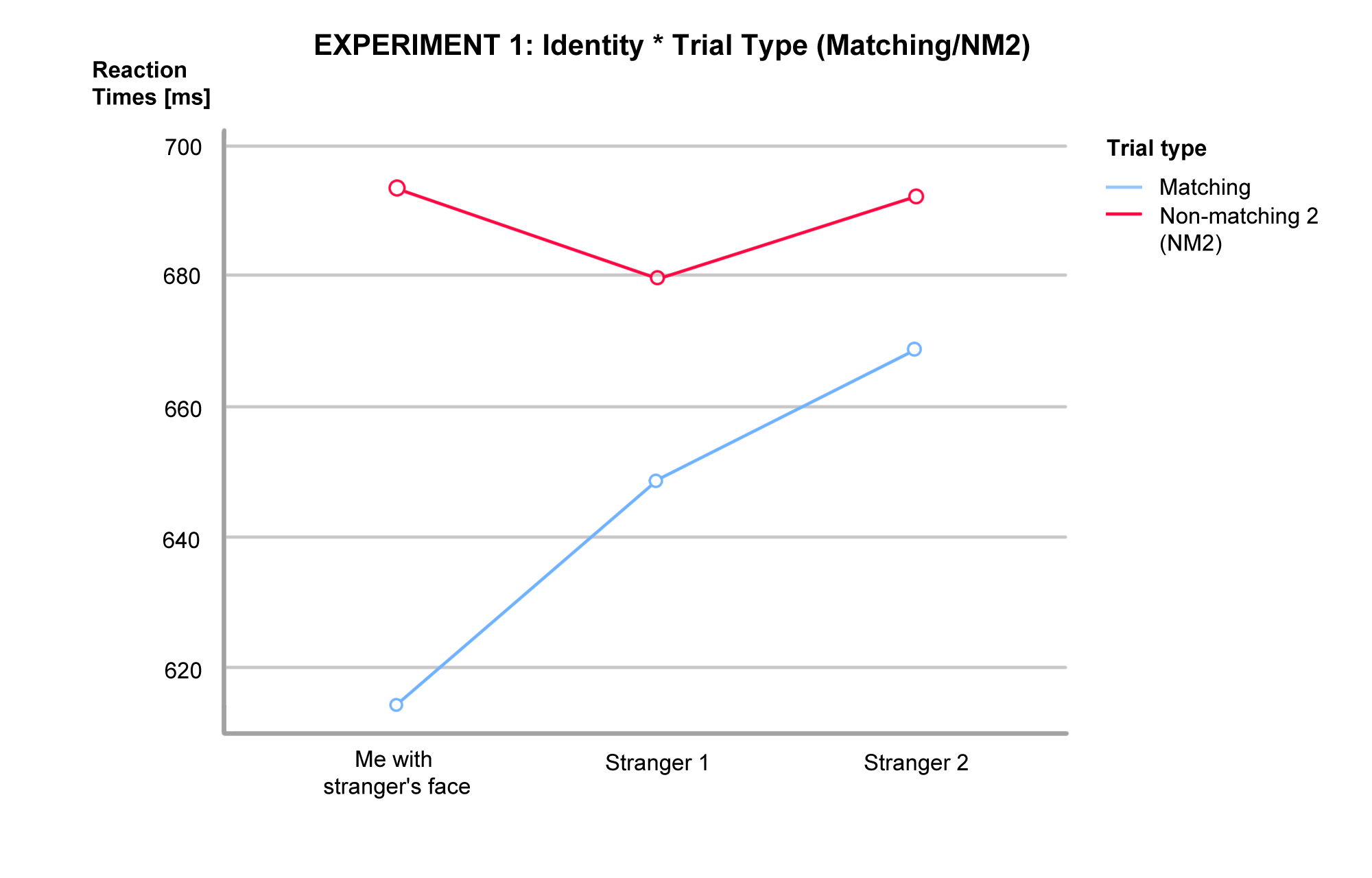


**Fig 2**. Average reaction times for matching and non-matching (NM2) trials for three identities (self-associated stranger’s face, stranger 1, stranger 2).

The results from additional analyses of experiment 1 replicate previous findings from Woźniak et al., (2018), who found that the NM1 mismatching trials exhibited the same pattern of results as the matching trials (hence, there was no interaction), while the NM2 trials showed lack of the effect present in the matching trials, which drove the interaction effect. These results are in agreement with the findings from the main analysis of experiment 1, i.e. that the self-related cue (label “You”) facilitates processing, but an unfamiliar self-associated target (a face matched with the self) does not. Moreover, it is worth noting that in NM2 trials processing of each identity of the target is differently affected by what the preceding cues are. For example, if the target in NM2 trial averaging is a self-associated face, then by necessity it must have been preceded by one of two stranger-associated labels. On the other hand, each stranger-related target is preceded 50% by a mismatching stranger-associated name, and 50% of the time by a self-related cue (label “You”). As a consequence, even if there is no difference between processing of self-related and non-self-related targets, one can still expect that non-self-related targets will be processed faster, because 50% of the time they are preceded by a self-associated cue which facilitates the “self-boost” effect, while self-related target never enjoys this benefit. This is exactly the pattern of results that has been reported by Woźniak et al., (2018), and which is present in our present results. It drives the interaction between identity and matching, and it is the direct reason why we adopted a different research design, and used a different method of analyzing the results than Woźniak et al.

**Experiment 2.**

**Matching vs non-matching 1 (NM1)**

In experiment 2, when contrasting matching trials with NM1 trials, main effects for both factors, trial type (*F*(1,23)=7.13, *p*=0.014, partial *η^2^*=0.24) and identity: (*F*(2,46)=9.10, *p*<0.001, partial *η^2^*=0.28), were statistically significant. The interaction between trial type and identity was also significant (*F*(2,46)=5.69, *p*=0.012, partial *η^2^*=0.20, GG-corrected). Post hoc HSD tests of the interaction effect revealed that the RTs for matching trials of “Stranger with my face” were faster than RTs to all other conditions (all *p* values < 0.01). No other differences were statistically significant.


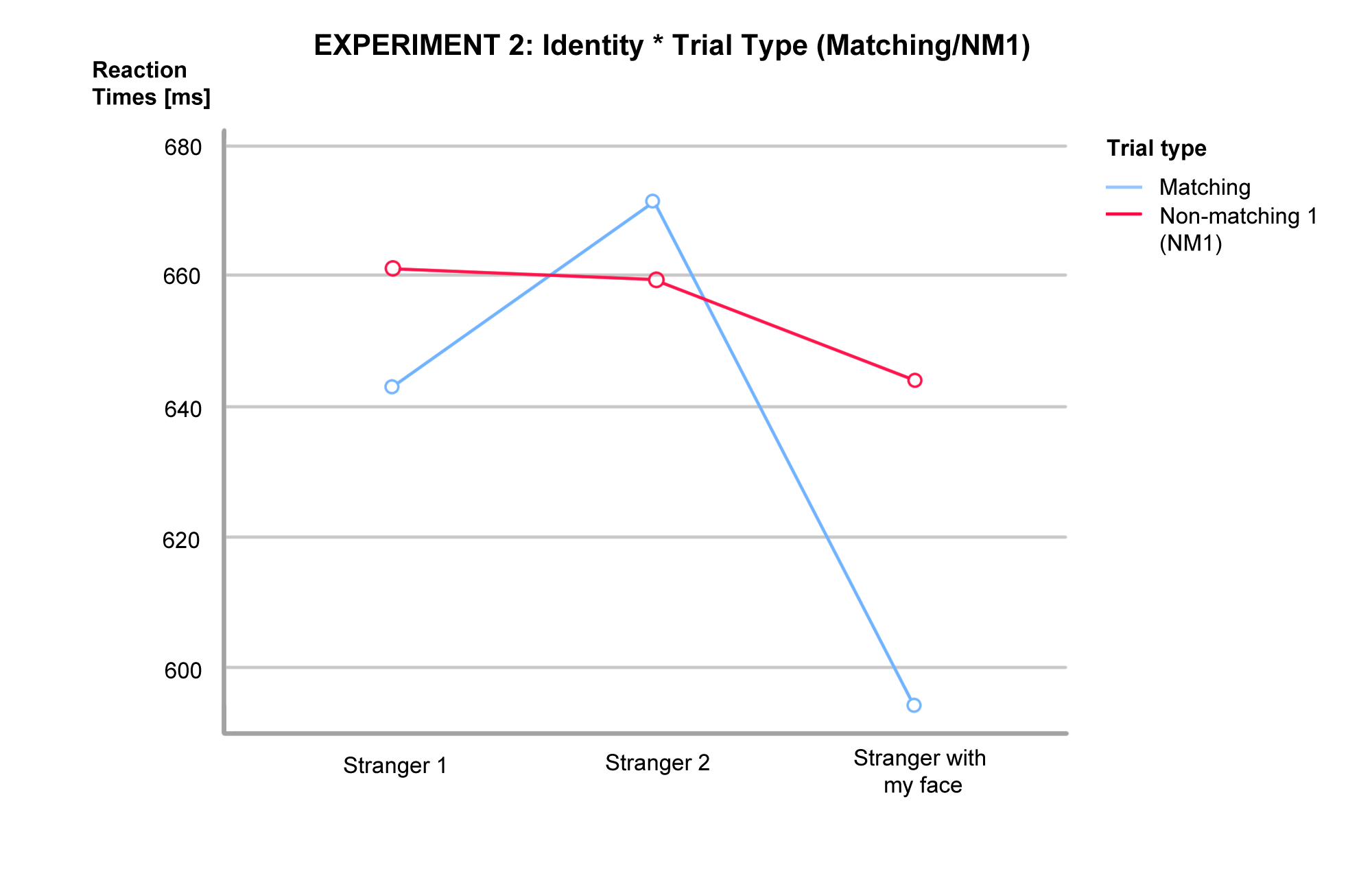


**Fig 3**. Average reaction times for matching and non-matching (NM1) trials for three identities (stranger 1, stranger 2, stranger-associated one’s real face).

**Matching vs non-matching 2 (NM2)**

The pattern of averages comparing the matching trials with NM2 trials was similar to the one present in NM1 trials, and both the main effect of trial type (*F*(1,23)=6.94, *p*=0.015, partial *η^2^*=0.23) and the main effect of identity (*F*(2,46)=13.17, *p*<0.001, partial *η^2^*=0.36, GG-corrected) were significant. Their interaction, however, did not exceed the threshold of significance (trial type*identity: *F*(2,46)=3.08, *p*=0.067, GG-corrected).


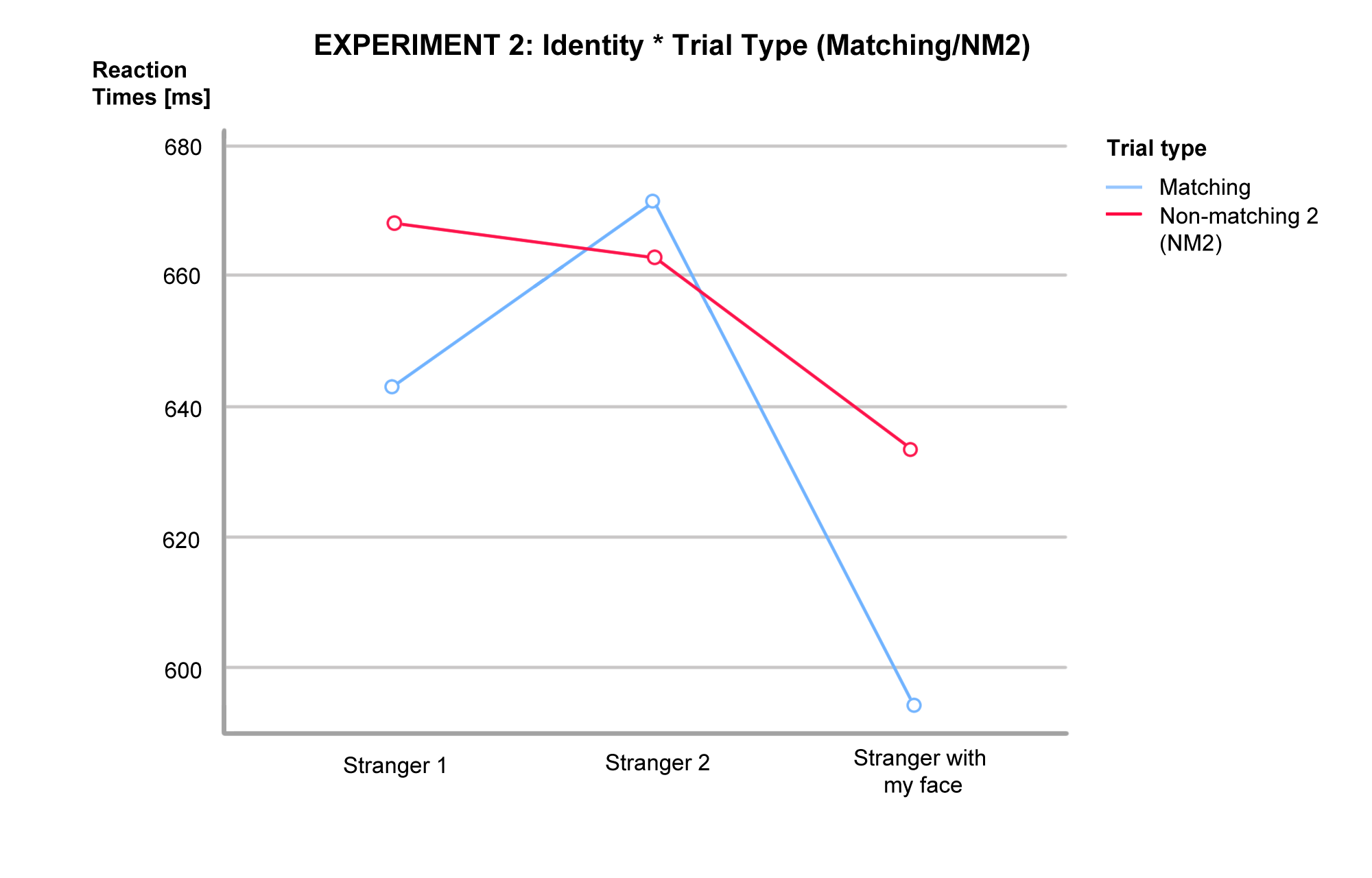


**Fig 4**. Average reaction times for matching and non-matching (NM2) trials for three identities (stranger 1, stranger 2, stranger-associated one’s real face).

Although the interaction effect for matching vs NM1 trials turned out to be significant, the overall pattern of results in experiment 2 was in agreement with our assumption. In both ANOVAs self-related conditions were processed faster than both stranger-identities. The significant interaction effect was driven by unusually slow reactions to the second stranger identity during the matching trials (and only matching trials) by three participants. The faces and names used as a second stranger’s identity in these trials were all different, so these outlier values cannot be caused by systematic problems with specific stimuli. Unfortunately, it was not possible to ask these participants if they knew why their responses were so slow, because data was not analysed immediately following the experimental procedure. Therefore, these results (and the significant interaction) can only reflect random fluctuations in the tested sample.

**Experiment 3.**

**Matching vs non-matching 1 (NM1)**

In experiment 3 the additional analysis contrasting matching with NM1 trials has shown a significant main effect of trial type (*F*(1,23)=24.01, *p*<0.001, partial *η^2^*=0.51) and significant interaction between trial type and identity (*F*(2,46)=3.35, *p*=0.044, partial *η^2^*=0.13). The main effect of identity was not significant (*F*(2,46)=0.76, *p*=0.98). Post hoc HSD tests on the interaction effect revealed that RTs in NM1 trials of “Stranger with my face” were significantly slower than RTs in the matching trials for “Stranger with my face” (*p*=0.49) and “Stranger” (*p*<0.001).


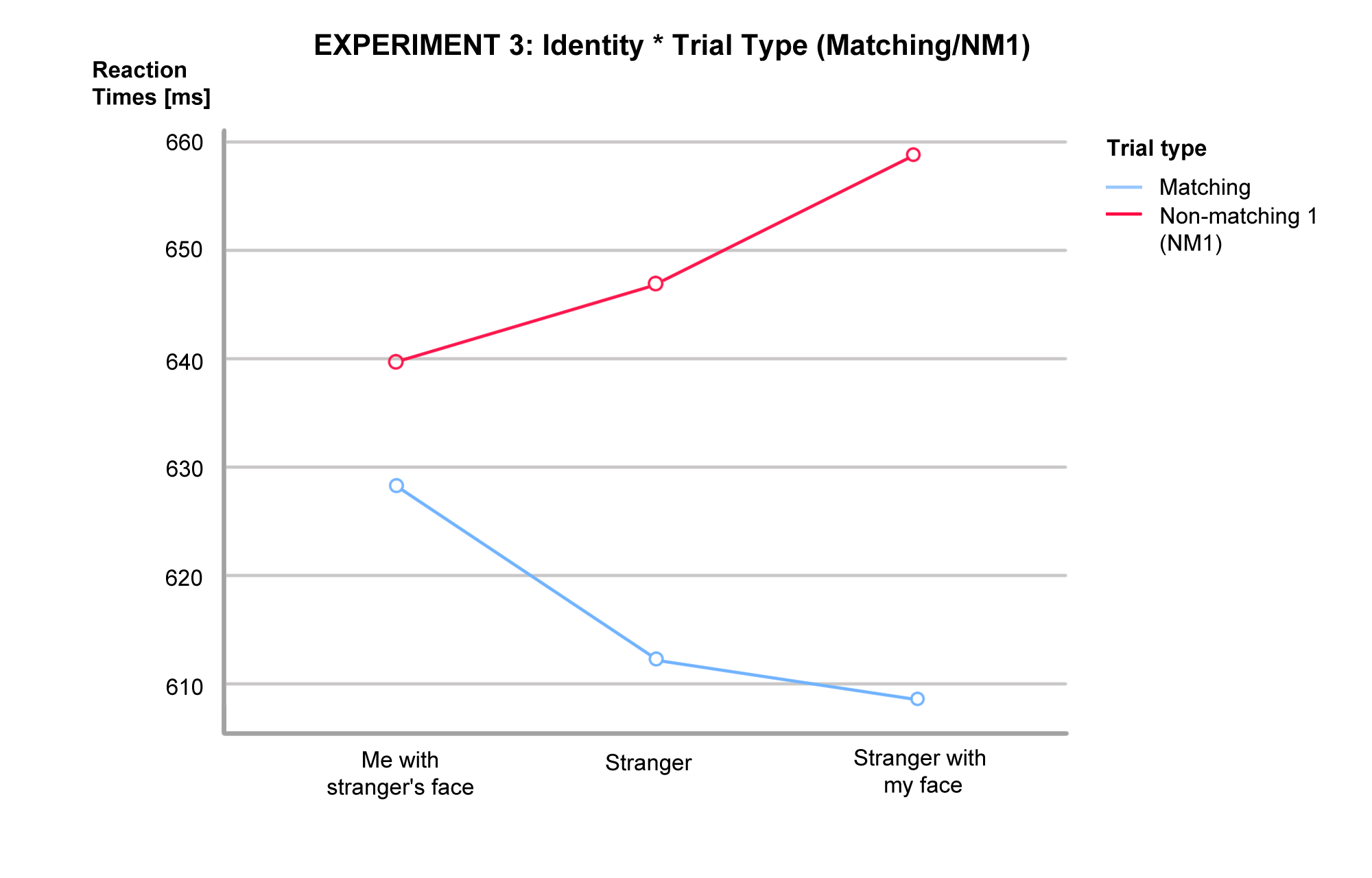


**Fig 5**. Average reaction times for matching and non-matching (NM1) trials for three identities (self-associated stranger’s face, stranger, stranger-associated one’s real face).

**Matching vs non-matching 2 (NM2)**

The analysis with NM2 showed standard pattern of results: the main effects of trial type (*F*(1,23)=24.16, *p*<0.001, partial *η^2^*=0.51) and identity (*F*(2,46)=5.2, *p*=0.009, partial *η^2^*=0.18) were both significant, but the interaction trial type*identity was not (*F*(2,46)=0.71, *p*=0.50).


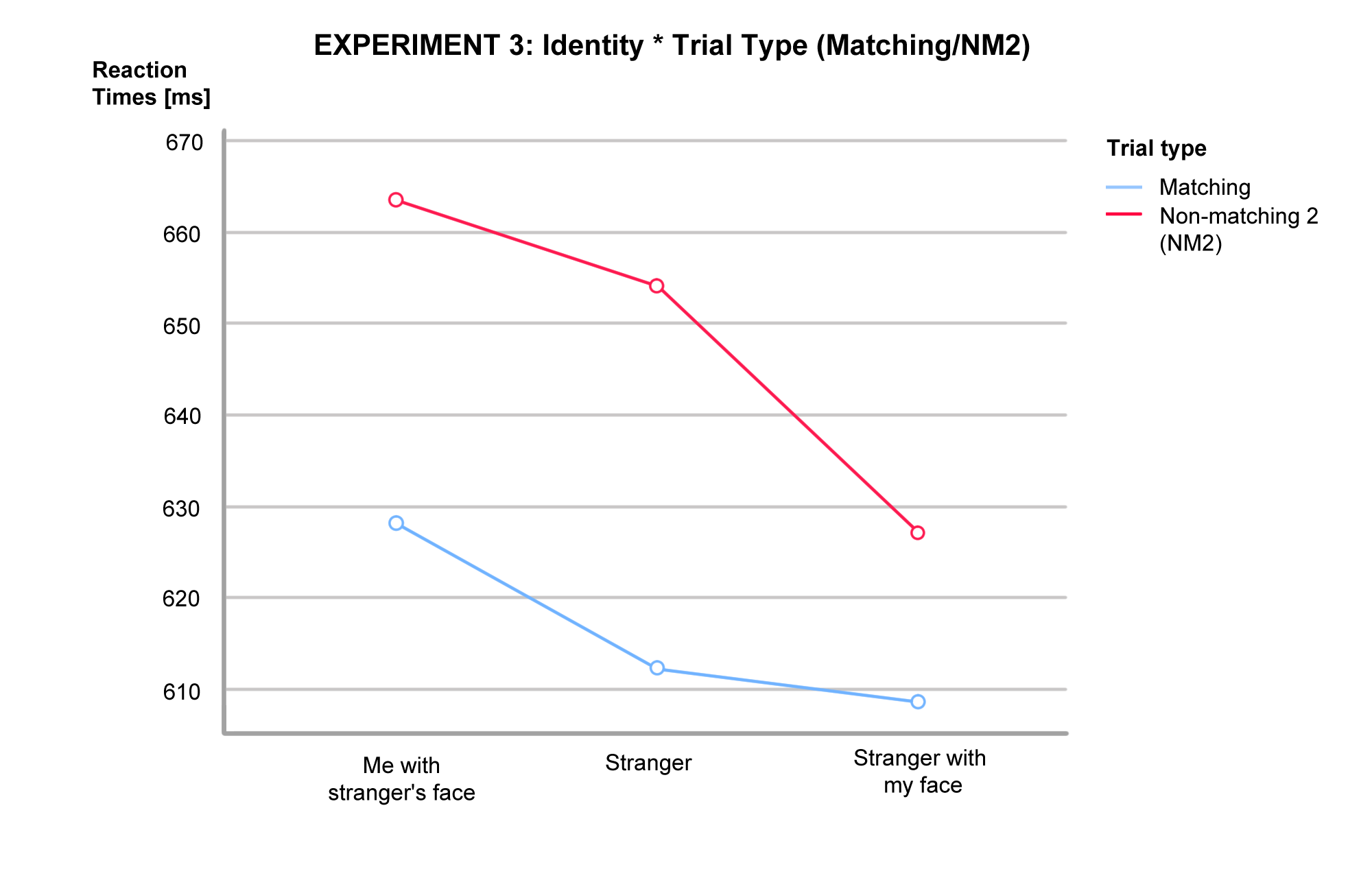


**Fig 6**. Average reaction times for matching and non-matching (NM2) trials for three identities (self-associated stranger’s face, stranger, stranger-associated one’s real face).

The results comparing matching trials with NM2 trials show no interaction between the two factors, although it is worth to note that in both the matching and mismatching pairs the slowest reaction times came following a self-associated stranger’s face. It suggests that presentation of that face may elicit a conflict, which leads to detrimental performance, regardless of whether it is present as a valid or invalid target. On the other hand, one’s real face was processed faster than other faces regardless of the type of trial, a result which is in line with the main analysis.

The results of analysis with the NM1 trials show a more complicated picture and supplement findings reported in the main body of the paper. Although the main effect of identity is absent, identity interacted with the trial type. In the matching trials presentation of the word associated with one’s real face led to the top-down self-prioritization effect, while presentation of the word “You” led to the slowest responses. This pattern, however, was reversed in NM1 mismatching trials, where the word “You” led to the fastest responses, and the name associated with one’s real face led to the slowest ones.

One way to interpret these results is by looking at the influence of the target stimulus on NM1 trials. As has been repeatedly found, also in our experiment 2, a picture of one’s real face is much easier to detect than other faces, especially when compared with unfamiliar faces of strangers. The problem with the NM1 way of calculating averages for mismatching trials (and the reason why we used a different method in the main analysis) is that if the cue is a label associated with a given target (e.g. “Meg”), then by necessity the trials with a valid target cannot be included in this average (i.e. a face associated with Meg). It means that for NM1 trials in which the cue has been associated with one’s real face (e.g. “Liz”) the target will consist of either a stranger’s face (e.g. Meg’s), or self-associated stranger’s face (associated with a cue “You”), which are both much more time-consuming to process than one’s real face. Conversely, if the cue is a label “You” or the name of a stranger (e.g. “Meg”) then during half of the mismatching trials one’s real face will be displayed, which is much easier to detect. As a consequence, it is expected that these trials will yield faster RTs, just because half of the time they are followed with much easier target to identify (one’s real face). Moreover, if the conclusions from analysis of the matching vs NM2 trials are correct, i.e. that in a situation of conflicting associations a self-associated face is more difficult to identify than other faces, then we can expect fastest reaction times in NM1 trials following the label “You”, just because it is followed by easier targets to identify. As such we would expect exactly the pattern of results which we observe, caused only by the imbalance in identity of the target stimuli following different cues.

This interpretation provides rationale for the decision to use a different method of analysing the data (and experimental design) in our study, than the one used in (Woźniak et al., 2018), where dividing the data into matching and mismatching trials led to less complications, because participants’ real faces weren’t used.

**References**

Woźniak, M., Kourtis, D., & Knoblich, G. (2018). Prioritization of arbitrary faces associated to self: An EEG study. *PLoS One, 13*(1), e0190679.
