# Supplementary material for: Stranger to my face: Top-down and bottom-up effects underlying prioritization of images of one’s face
Source: PLoS One. 2020 Jul 9;15(7):e0235627. doi: 10.1371/journal.pone.0235627 (PMC7347180; doi:10.1371/journal.pone.0235627)
Supplement: S2 Text — (DOCX) [file pone.0235627.s002.docx]

**S2 Text. Supplementary figures illustrating Cue X Target interaction effects.**

The supplementary figures illustrate interaction effects between cue- and target-associated identities for each of three experiments. In all figures grey circles indicate matching trials. Grey rectangles illustrate trials preceded by self-associated cues in Experiment 1 and Experiment 2. As can be seen, matching trials are associated with faster reaction times (especially in trials which involve self-associated cues) and decreased accuracy.

**Experiment 1.**


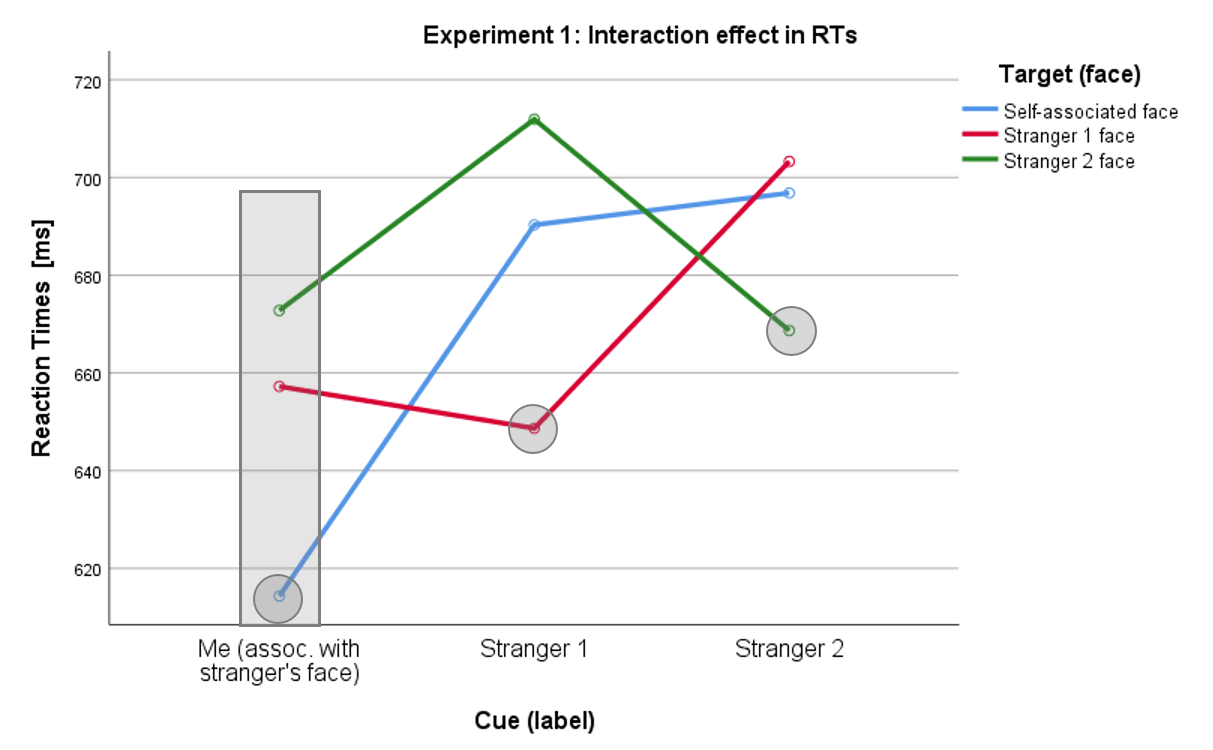


**Fig 1**. Average reaction times in Experiment 1 across all types of trials.


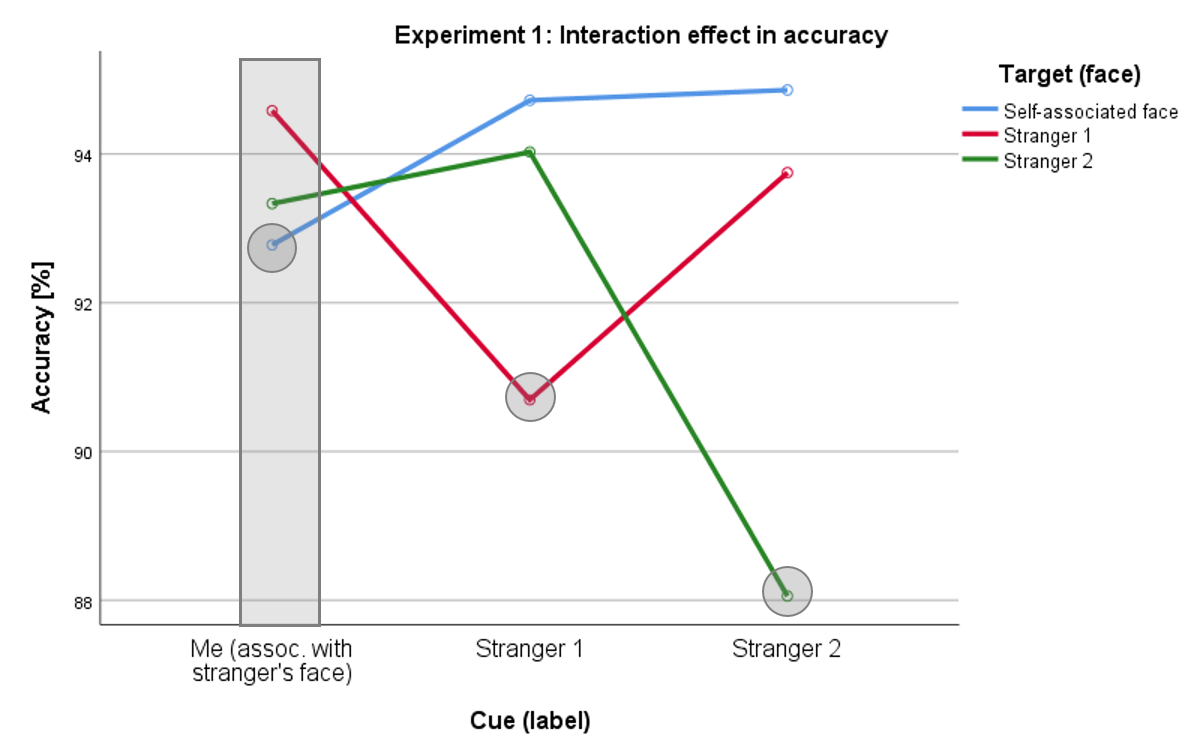


**Fig 2**. Accuracy in Experiment 1 across all types of trials.

**Experiment 2.**


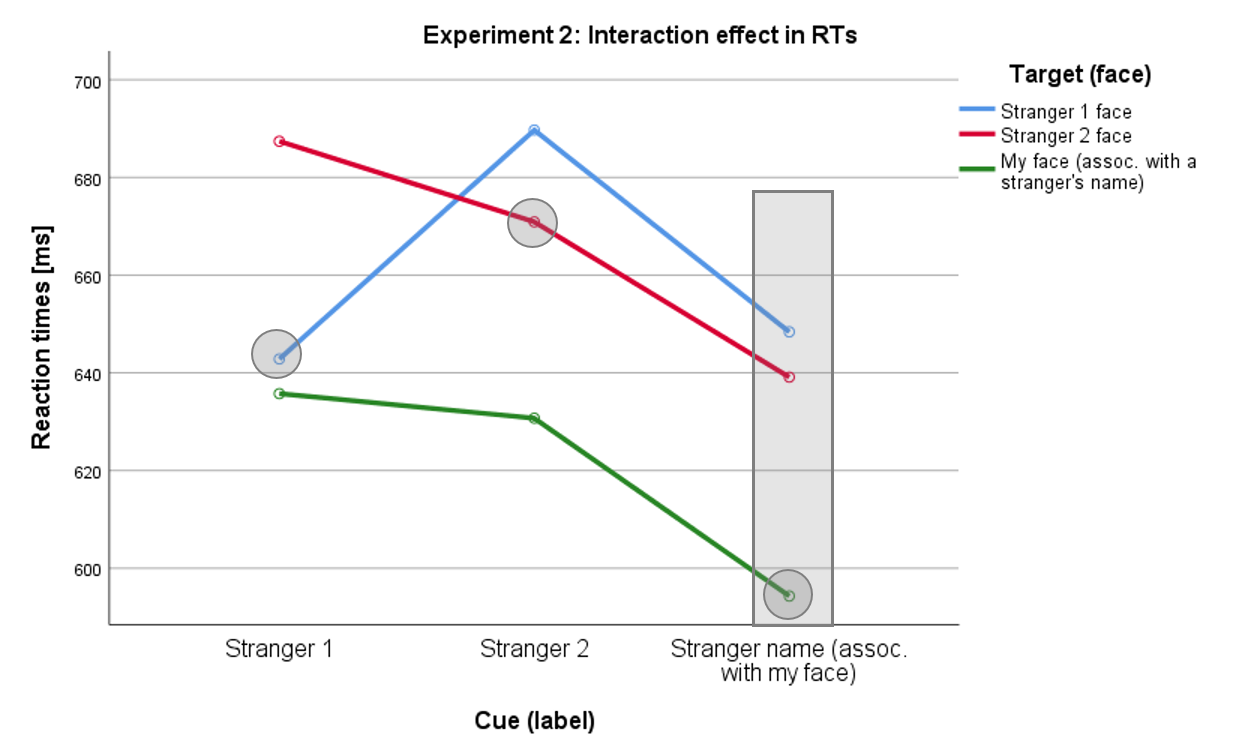


**Fig 3**. Average reaction times in Experiment 2 across all types of trials.


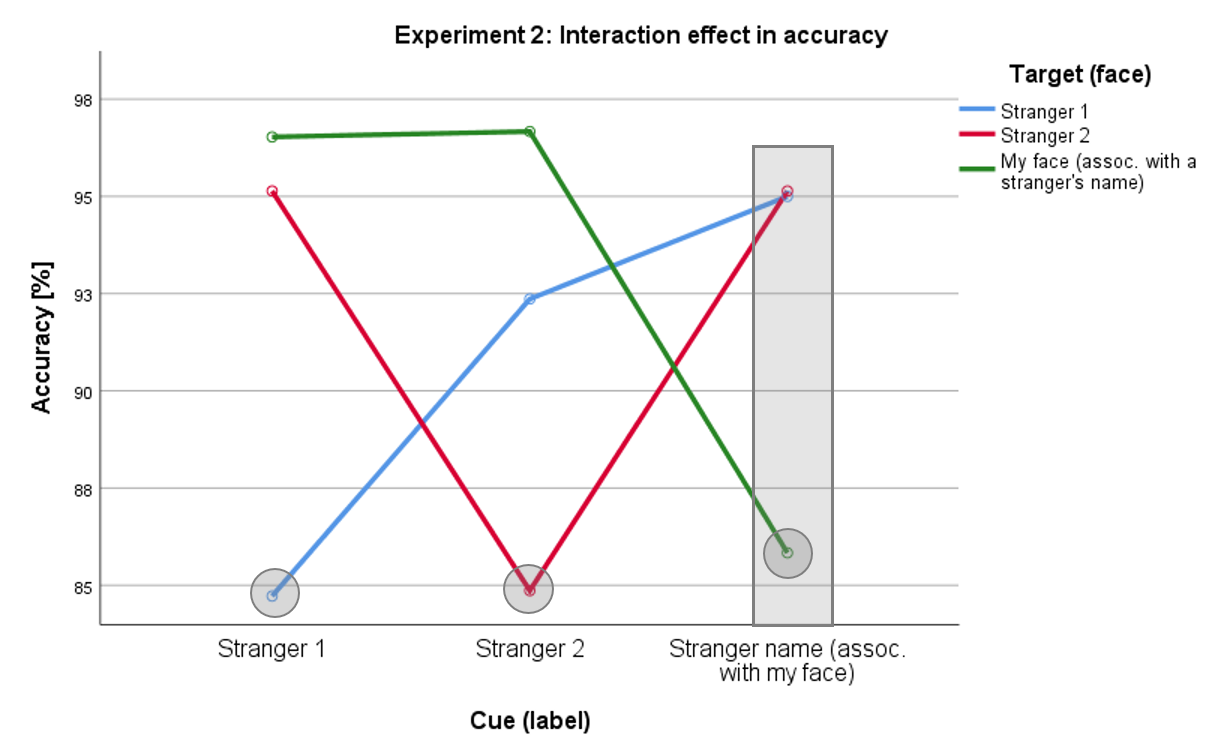


**Fig 4**. Accuracy in Experiment 2 across all types of trials.

**Experiment 3.**


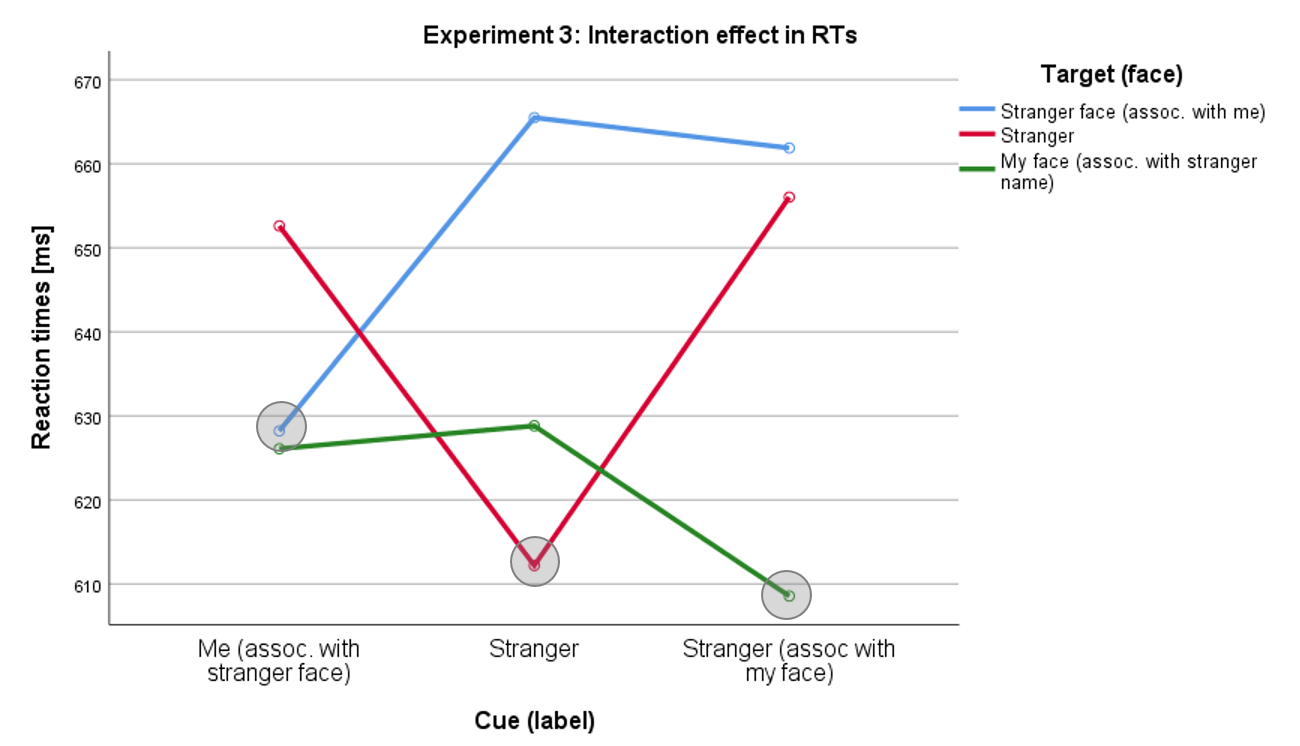


**Fig 5**. Average reaction times in Experiment 3 across all types of trials.


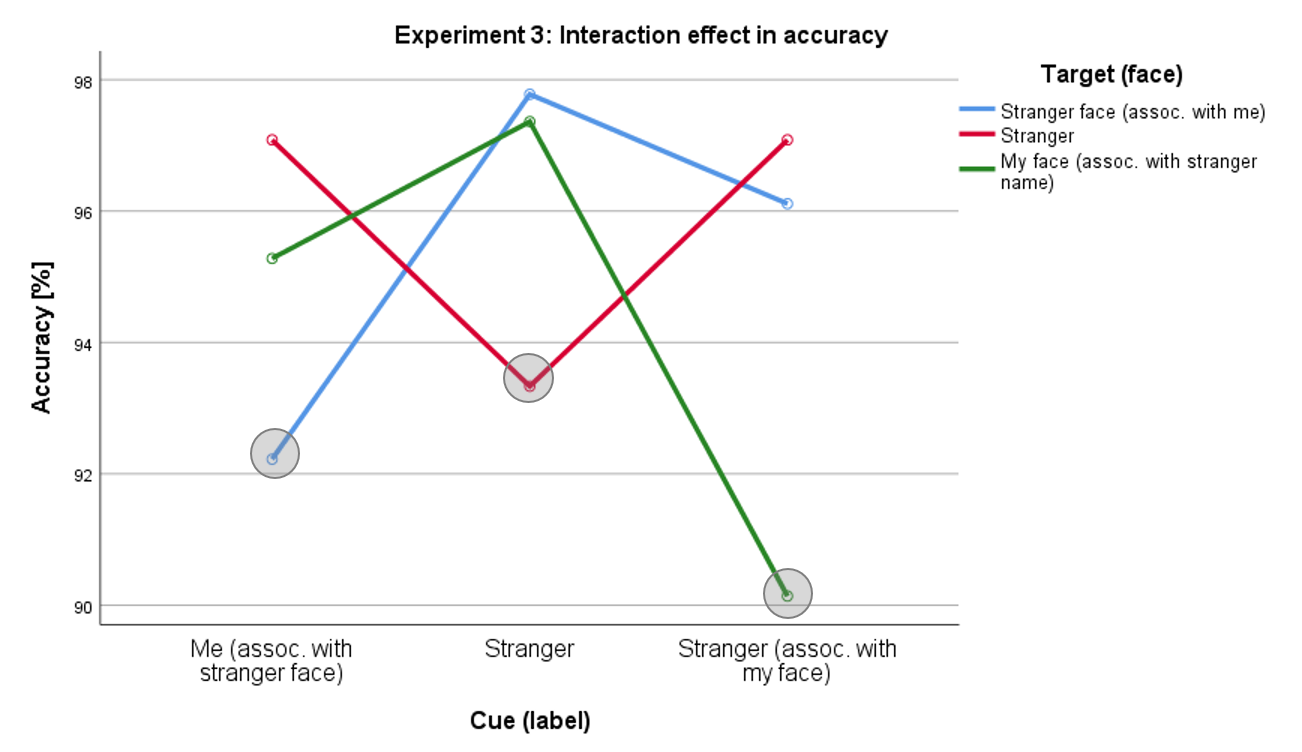


**Fig 6**. Accuracy in Experiment 3 across all types of trials.
